# Nurses’ and midwives’ knowledge and safe-handling practices related to hazardous drugs: A cross-sectional study

**DOI:** 10.1016/j.ijnsa.2025.100331

**Published:** 2025-04-14

**Authors:** Pheona van Huizen, Paul Wembridge, Philip L. Russo, Elizabeth Manias, Clifford J. Connell

**Affiliations:** a Monash Nursing and Midwifery, Monash University, Australia; b Clinical Risk Manager (Medication Safety), Eastern Health, Victoria, Australia; c Cabrini Research, Cabrini Health, Australia

**Keywords:** Hazardous drugs, Hierarchy of controls, Knowledge, Medications, Midwife, Nurse, Occupational exposure risk, Personal protective equipment, Safe handling practice

## Abstract

**Background:**

Hazardous drugs are inherently toxic and can present an occupational exposure risk to healthcare professionals. Hazardous drugs are regularly prescribed for people to treat a variety of medical conditions.

**Aim:**

To explore nurses’ and midwives’ knowledge and practices related to the safe handling of hazardous drugs. Also, to discover if controls of risk are available to support nurses and midwives to implement best practices when handling hazardous drugs in health care settings and if there are any obstacles to using these controls.

**Method:**

This was a cross-sectional study using a convenience sample of nurses and midwives at six Australian hospitals from a metropolitan health care service. In the self-administered, validated online questionnaire, participants were asked to identify hazardous drugs and their use of hazard controls, including personal protective equipment. Variables for self-efficacy, perceived barriers, perceived risk, interpersonal influences, and workplace safety climate were also measured.

**Results:**

A total of 217 nurses and midwives reported they handled hazardous drugs. The questionnaire was completed in full by 156 participants (71.9 %). Participants predominantly identified as women (196/217, 90.3 %) and had completed a bachelor's of nursing (139/217, 64.1 %). The hazardous drugs chloramphenicol, colchicine, and dutasteride were frequently not identified as hazardous (80.6 %, 73.8 %, and 68.2 %, respectively). A total of 78 (35.9 %) participants reported having received hazardous drug handling training, of which 34 (43.6 %) stated it was in the past 12 months. Participants (181/203, 89.2 %) agreed or strongly agreed that they were confident that they could use personal protective equipment correctly and were provided with the best available personal protective equipment (163/203, 80.3 %). Despite this, personal protective equipment was never worn by approximately a third of participants who reported being involved in preparing, administering, and disposing of hazardous drugs (32.2 %, 29.8 %, and 30.9 %, respectively).

**Conclusion:**

Nurses and midwives did not always recognise hazardous drugs, and, although they were confident in using appropriate personal protective equipment, some reported never wearing it. A multi-faceted multidisciplinary intervention is needed to improve both knowledge and practice for handling both cytotoxic and non-cytotoxic drugs by nurses and midwives.

**What is already known**

• Nurses working in cancer services do not always know there is a potential risk of occupational exposure from handling hazardous drugs, and, even when they do, they do not always wear the recommended personal protective equipment.

**What this paper adds**

• Nurses and midwives working throughout a healthcare service in Australia did not always recognise hazardous drugs as an occupational hazard.
• Twice as many nurses and midwives reported wearing personal protective equipment when handling hazardous injectables drugs compared to oral formulations.
• Approximately one-third-of nurses and midwives reported that they never wore personal protective equipment when preparing, administering, or disposing of injectable and oral hazardous drug formulations.

## 1 Background

The term hazardous drug was first used in 1990 by the American Society of Hospital Pharmacists. The purpose of this term was not to restrict the relationship between the risk of occupational exposure from a drug to its intended use or classification, such as anticancer or cytotoxic, but to be more inclusive of agents that continue to be developed and agents that we learn are hazardous in nature (American Society of Hospital Pharmacists ASHP, 1990). In a comprehensive and often cited revision of the American Society of Hospital Pharmacists’ definition by the United States National Institute for Occupational Safety and Health, a hazardous drug is defined as having one or more of the following toxicity criteria: carcinogenicity, teratogenicity or other developmental toxicity, reproductive toxicity, organ toxicity at low doses, or genotoxicity (National Institute for Occupational Safety and Health, 2024). A hazardous drug has also been defined as “a drug whose inherent toxicity presents a danger to healthcare personnel” (International Society of Oncology Pharmacy Practitioners Standards Committee, 2022, p.3).

There is clear evidence demonstrating risk from the occupational exposure of nurses to hazardous cytotoxic drugs, despite the use of control measures to mitigate this risk (Delafoy et al., 2023; Gruenewald and Gilkey, 2021; Kennedy et al., 2023). It has been reported that nurses do not always know about the potential for occupational exposure from cytotoxic drugs and, even when they do, there is a wide variation in safe handling practices (Pirot et al., 2023; Asefa et al., 2021; van Huizen et al., 2024a). In contrast to cytotoxic hazardous drugs, there is scant research reporting on the risk from non-cytotoxic hazardous drugs and what nurses outside of specialist oncology settings know and do about their risk of occupational exposure. Furthermore, there are no known studies about midwives regarding their knowledge and use of hazardous drugs (van Huizen et al., 2024a).

### 1.1 Pender's health promotion model

The original Pender's Health Promotion model was adapted by Lusk et al. (1997) for use in occupational health research. It was further adapted by Polovich and Clark (2012) from a study about the use of hearing protection (Raymond et al., 2006). In the study by Polovich and Clark (2012), Pender's Health Promotion model was used as a theoretical framework for predicting factors that influence the use of hazardous drug safe-handling practices. In this study, the revised Pender's Health Promotion Model was used to explore factors directly influencing the safe handling practices for hazardous drugs by nurses and midwives.

The revised Pender's Health Promotion model consists of three components: individual characteristics and experience, behaviour specific cognitions and affect, and behavioural outcome (Pender et al., 2015). The variables collected, to evaluate the factors directly influencing the safe handling practices for hazardous drugs in the revised Pender's Health Promotion Model, are shown in Figure 1 in the Supplementary Material.

### 1.2 Aim

The primary aim of this study was to explore nurses’ and midwives’ knowledge and practices related to the safe handling of hazardous drugs. A secondary aim was to discover if controls of risk were available to support nurses and midwives to implement best practices when handling hazardous drugs in health care settings and if there were any obstacles to using these controls.

## 2 Method

### 2.1 The population and sample

The study was conducted in the second largest of Victoria, Australia's 18 public healthcare services. The population included were registered and medication-endorsed enrolled nurses and midwives who had administered drugs to patients in any clinical area of six hospitals from the metropolitan healthcare service.

Convenience sampling was used to recruit participants at the healthcare service's sites. The aim of the recruitment strategy was to minimise sampling bias with representativeness closely approximating the population of nurses and midwives with a clinical workload in each healthcare area. A minimum sample size of 123 was calculated using the G*Power software (version 3.1.9.4) to understand the relationships among the predictor variables (moderate correlation of 0.25 as the alternative hypothesis, *α* significance level of <0.05, and power 0.80) (Faul et al., 2009).

The clinical nurse educators responsible for each healthcare area were given posters and postcards to advertise the project and distribute to eligible staff. During the study period, the clinical nurse educators received a weekly email containing an update of the number of participants recruited and an educational tip related to hazardous drugs. These updates served to remind educators to continue to encourage nurses and midwives to participate. The questionnaire was open for 16 weeks from the 9th of January until the 30th of April 2023.

### 2.2 The study instrument

This was a cross-sectional study, utilising a self-administered online validated questionnaire. Prior to modifying the ‘Revised hazardous drug handling questionnaire’ (Polovich and Clark, 2012) for use, permission was obtained from the instrument developers. Polovich and Clark's questionnaire incorporated scales from various sources (Supplementary Material) and was originally used with a sample of 165 oncology nurses across the United States.

The ensuing ‘What nurses and midwives know about and do when handling hazardous drugs’ questionnaire was published on the Qualtrics web-based platform and was accessible by a QR code (Qualtrics, 2022). The QR code was the same for all participants, ensuring the anonymity of each response. Due to the length of the questionnaire, it is unlikely that each participant would have completed the questionnaire more than once. The participant information and consent form were located at the beginning of the questionnaire, and participation in the questionnaire implied informed consent.

The adaptation of the *Revised hazardous drug handling questionnaire* for this study enabled the comparison of the dependent and independent variables with the data from previous studies. Modifications included rewording some items and the addition of questions so that the questionnaire could be used with a population locally in Victoria, Australia. The Likert scales for *self-efficacy, perceived barriers, perceived risk*, and *conflict of interest* were also changed to a 5-point scale to match the *workplace safety climate* scale. This streamlined the scoring scales for participants, making the questionnaire more cohesive and capturing additional variability. The population was a heterogeneous convenience sample of nurses and midwives across the healthcare service and was not limited to those working in cancer services. These modifications and the use of the questionnaire with a different population meant that the validity and reliability were established before use; see Supplementary Material.

### 2.3 Measures

The final version of the questionnaire comprised 44 questions. The questions included nine scales measuring variables related to the revised Pender's Health Promotion Model (Supplementary Material). Additionally, there were ‘use of personal protective equipment’ variables for the preparation, administration, disposal, and handling of bodily fluids, with separate scales for cytotoxic and non-cytotoxic hazardous drugs (Supplementary Material).

### 2.4 Data source, use and statistical analysis

Data were stored on the secure university network and imported into IBM SPSS Statistics version 28 (IBM Corp., Armonk NY, USA). The number of participants in the sample who completed questions as they moved through the questionnaire was reported using flowcharts (Supplementary Material).

Prior to addressing the research question, data were examined for normal distribution, any patterned response sets (looking for identical or patterned responses in all items, including the omission of most items), and outliers (Cronbach, 1946). If patterned response sets were identified, the data were excluded.

Descriptive statistics were used to analyse and present the demographic and item results in the first level of analysis. The descriptive statistics included calculations of frequency distributions, measures of central tendency and means, standard deviations (SDs), or interquartile ranges as appropriate. Multi-scale items from the questionnaire had total scale scores calculated. The Cronbach's alpha was calculated for the handling activities for both cytotoxic and non-cytotoxic hazardous drugs and for each of the scale variables.

At the second level of analysis, the theoretical predictor variables influencing the use of personal protective equipment for the safe handling of hazardous drugs were plotted and evaluated for normality and outliers. Spearman's rho correlation coefficients were calculated for the continuous variables, and independent samples *t*-tests were used to find differences. Correlations and differences between variables were calculated to determine the relationship between nurses’ use of personal protective equipment when handling hazardous drugs and the revised Pender's Health Promotion Model predictor variables of *knowledge about risk of exposure, self-efficacy for using personal protective equipment, barriers to using personal protective equipment*’, *perceived risk, conflict of interest, interpersonal modelling, interpersonal norms*, and *workplace safety climate*, as well as years of nursing experience, level of education, and ward or clinical area being dedicated to cancer services (*p*<.05).

### 2.5 Risks and ethical issues

A low-risk ethics approval was granted from the health service and then Monash University (LR22–053–89,944, project number 36,369).

## 3 Results

The questionnaire was started by 310 nurses and midwives. Of these, five questionnaires were blank, and 54 participants completed only the demographics section. Due to the structure of the questionnaire, with skip logic used in Qualtrics for some questions and the time required to complete all 44 questions, 156 participants completed the total questionnaire. (There is a flowchart illustrating this in the Supplementary Material.)

It was not possible to calculate a true response rate for the questionnaire due to the recruitment strategy used. This approach resulted in a denominator of the number of nurses and midwives who knew about the study being incalculable.

### 3.1 Nurse and midwife demographics

Participants were asked if they handled hazardous drugs after the drug identification question. This was deliberate to demonstrate that some routinely administered medications could be classified as hazardous drugs. The participants who reported handling hazardous drugs (*n* = 217) predominately identified as women. Approximately half were aged between 20 and 34 years and had one to ten years of nursing or midwifery experience. The majority of participants had completed a Bachelor's of Nursing degree (Table 1).

**Table 1:** Demographic data for nurses and midwives who reported they do not and do handle hazardous drugs in their workplace.

	Category	Do not handle hazardous drugs (*n* = 32)	Percentage ( %)	Handle hazardous drugs (*n* = 217)	Percentage ( %)
Gender	Woman	32	100.0	196	90.3
	Man	-	-	18	8.3
	Non-binary/gender diverse	-	-	2	0.9
	Prefer not to say	-	-	1	0.5
					
Age	20–29	10	31.3	67	30.9
	30–39	6	18.8	81	37.3
	40–49	5	15.6	35	16.1
	50–59	6	18.8	23	10.6
	60 or older	5	15.6	11	5.1
					
Education completed*	Diploma of Nursing	3	9.4	39	18.0
	Bachelor's of Nursing	20	62.5	139	64.1
	Bachelor's of Nursing (Honours)	1	3.1	6	2.8
	Bachelor's of Midwifery	4	12.5	17	7.8
	Graduate Certificate of Nursing	4	12.5	43	19.8
	Graduate Diploma of Nursing	2	6.3	16	7.4
	Master's of Nursing	2	6.3	16	7.4
	PhD	-	-	-	-
	Other	-	-	17^a^	7.8
					
Nursing experience	<1 year	3	9.4	16	7.4
	1–5	6	18.8	62	28.6
	6–10	4	12.5	50	23.0
	11–15	5	15.6	34	15.7
	16–20	3	9.4	15	6.9
	21–25	2	6.3	12	5.5
	26 or more	9	28.1	28	12.9
					
Employment	Full-time	8	25.0	47	21.7
	Part-time	22	68.8	159	73.3
	Casual	2	6.3	11	5.1
					
Work setting	Inpatient acute ward	21	65.6	147	67.7
	Inpatient sub-acute ward	3	9.4	46	21.2
	Day haematology or oncology	-	-	5	2.3
	Outpatient and same day settings	3	9.4	10	4.6
	Residential care	2	6.3	8	3.7
	Operating theatre	3	9.4	1	0.5
					
Healthcare area	Cancer services	-	-	20	9.2
	Cardiology	-	-	7	3.2
	Emergency	1	3.1	26	12.0
	Geriatric medicine	7	21.9	41	18.9
	Intensive care services	1	3.1	16	7.4
	Maternity services	4	12.5	16	7.4
	Medical	11	34.4	32	14.7
	Mental health services	3	9.4	3	1.4
	Paediatrics	-	-	3	1.4
	Palliative care	2	6.3	7	3.2
	Surgical	3	9.4	45	20.7
	Unspecified	-	-	1	0.5

⁎ **Participants were able to select more than one type of education**. Education other^a^: Training and assessment (Technical and Further Education Certificate IV) (n = 1), Bachelor of public health and health promotion (n = 1), hospital trained (n = 3), third year Bachelor of nursing student employed as a Registered Undergraduate Student of Nursing (RUSON) and already qualified as an enrolled nurse (n = 1), post graduate in dementia care and nurse immuniser course (n = 1), Aged care (Technical and Further Education Certificate III) and Infection prevention and control course (n = 1), unspecified (n = 9).

In all, 252 nurses, five midwives, and 16 participants who were both nurses and midwives completed the ‘hazardous drugs identification’ question. The participants scored a mean of 7.6 (SD 2.41) correct responses out of a possible 14. Acetylcysteine (also known as N-acetylcysteine or NAC) and dutasteride were the drugs that had the highest number of *Don't know* responses. The drugs with the most correct responses were paracetamol, methotrexate, loratadine, and ibuprofen (Table 2).

**Table 2:** ‘Hazardous drug identification’ results (*n* = 252).

Table notes: Scoring: Correct response (shaded) = 1, all other responses (unshaded) = 0

### 3.2 The types of hazardous drugs handled by participants

Of the 217 participants who responded that they did handle hazardous drugs at work, 17 (7.8 %) reported they handled cytotoxic drugs only, 93 (42.9 %) reported handling hazardous non-cytotoxic drugs only, and 106 (48.8 %) nurses and midwives reported they handled both cytotoxic and non-cytotoxic hazardous drugs (1 missing response) (Supplementary Material).

### 3.3 Education and training (*n* = 217)

In all, 94 (43.4 %) participants reported receiving no training in handling hazardous drugs, 78 (35.9 %) received training, and 44 (20.3 %) were unsure (*n* = 217, 1 missing response). For those who had received training (*n* = 78), 34 (43.6 %) participants stated it was in the past 12 months, 17 (21.8 %) two years since training, 14 (17.9 %) training was three or more years ago, and 6 (7.7 %) were unsure (7 missing responses).

### 3.4 The administrative and personal protective equipment hazard controls: Policies, guidelines, and the availability of personal protective equipment (*n* = 203)

When asked if their workplace had written policies and guidelines for handling hazardous drugs (*n* = 203), 135 (66.5 %) participants responded *Yes*, 8 (3.9 %) responded *No*, and 60 (29.6 %) didn’t know. The personal protective equipment available for performing hazardous drug handling activities included gloves (195 [96.1 %]), gowns or aprons (178 [87.7 %]), eye protection (181 [89.2 %]), and N95 or P2 particulate filter masks (189 [93.1 %]) (5 missing responses). Participants (198 [97.5 %]) reported that N95 or P2 face masks were expected to be worn in all clinical healthcare areas because of the COVID-19 pandemic.

#### 3.4.1 Identifying avoidable sources of environmental contamination

In response to the question “Before, during, or at the end of your nursing shifts, do you ever need to count drugs by using a pill counting tray?”, 148 (72.9 %) participants responded ‘Yes’ (*n* = 203), 1 missing response.

### 3.5 Preparation practices and storage (*n* = 178)

Hazardous drugs were reported by participants to be prepared either in the onsite pharmacy (95 [53.4 %]), off-site and delivered to the infusion area (34 [19.1 %]), in a separate drug storage and preparation room (92 [51.7 %]), the patient treatment area (49 [27.5 %]), or on a drug storage trolley in the hallway of the healthcare area (51 [28.7 %]) (*n* = 178); more than one answer could be selected, 20 missing responses). Participants reported that when they removed tablets or capsules from packaging, they used an aseptic non-touch technique with no gloves (94 [52.8 %]), used aseptic non-touch technique with gloves (32 [18.0 %]), used standard handling (touching if necessary) of tablets with gloves (11 [6.2 %]), or standard handling of tablets without gloves (38 [21.3 %]) (*n* = 178), 1 missing response.

Participants reported that when they needed to crush a drug to prepare it for administration, the drug was crushed using a mortar and pestle (106 [59.6 %]), a pill crushing device (37 [20.8 %]), in a syringe (2 [1.1 %]) or dispersed in water (8 [4.5 %]), and other: used both a pill crushing device and a mortar and pestle (3 [1.7 %]) (*n* = 178), 22 missing responses.

To check if a drug should be crushed, 102 (57.3 %) would ask a pharmacist, 120 (67.4 %) would use the ‘Don't rush to crush’ handbook (Symons and Ermer, 2021: evidence-based guide produced by The Society of Hospital Pharmacists of Australia ), 112 (62.9 %) would use MIMS (Monthly Index of Medical Specialties: information from pharmaceutical companies) (Leilani Au, July 2023), 43 (24.2 %) would ask a more senior nurse, 2 (1.1 %) would look for warnings in the electronic Medication Administration Record, and 1 (0.6 %) would crush the drug if the tablet was scored (*n* = 178); more than one response could be selected; 12 missing responses.

### 3.6 Engineering and personal protective equipment hazard controls

In this sample, participants reported that they were not involved in all hazardous drug handling activities (Table 3). The highest proportion of participants who answered these questions reported being involved in the administration of hazardous drugs. When involved, participants chose to wear different personal protective equipment depending on whether they were handling injectable hazardous drugs compared to oral formulations; some reported not wearing personal protective equipment for handling activities (Table 3). The use of personal protective equipment by participants when they handled injectable hazardous drugs was approximately double compared to the proportion of participants handling oral formulations.

**Table 3:** The use of personal protective equipment by participants for different hazardous drug handling activities and different dosage formulations, *n* ( %).

	Preparation *n* = 196		Administration *n* = 177		Disposal *n* = 164		Handle bodily fluids *n* = 163	
Perform handling activity	No	Yes	No	Yes	No	Yes	No	Yes
*n* ( %)	44 (22.4)	152 (77.6)	16 (9.0)	161 (91.0)	28 (17.1)	136 (82.9)	20 (12.3)	143 (87.7)
Wear personal protective equipment for handling activity?		*n* = 152		*n* = 161		*n* = 136		*n* = 143
Handle bodily fluids *n* ( %)								125 (87.4)
Injectables*n ( %)		97 (63.8)		104 (64.6)		87 (64.0)		
Oral formulations*n ( %)		47 (30.9)		51 (31.7)		50 (36.8)		
Never *n* ( %)		49 (32.2)		48 (29.8)		42 (30.9)		18 (12.6)

⁎ More than one response could be selected.

The data for the use of engineering and personal protective equipment hazard controls by participants handling cytotoxic and non-cytotoxic hazardous drugs when performing each of the four hazardous drug handling activities (preparation, administration, disposal, and bodily fluids) are summarised in the Supplementary Material. A greater number of participants reported handling non-cytotoxic hazardous drugs than cytotoxic hazardous drugs for each activity. The use of chemotherapy gloves and gowns was reported to be higher for handling cytotoxic than for non-cytotoxic hazardous drugs, and the use of N95 or P2 particulate filter masks was at least 80 % for all handling activities with both cytotoxic and non-cytotoxic hazardous drugs.

The score for personal protective equipment use for each handling activity was defined as the median of the total scores for the use of double gloves, impermeable gown, eye protection and N95 or P2 particulate filter mask for cytotoxic hazardous drugs; and gloves (highest score any type), impermeable gown, eye protection, and N95 or P2 particulate filter mask for non-cytotoxic drugs. The ‘total personal protective equipment use’ score was calculated for each of the 40 participants who reported they performed all four handling activities when handling cytotoxic hazardous drugs and the 54 participants who reported performing all four handling activities with non-cytotoxic hazardous drugs (Table 4). The Victorian Therapeutics Advisory Group guideline for the recommended personal protective equipment, which was used as the basis for these variables, is summarised in the Supplementary Material.

**Table 4:** The use of personal protective equipment for different handling activities for cytotoxic and non-cytotoxic hazardous drugs.

	Preparation personal protective equipment use	Administration personal protective equipment use		Disposal personal protective equipment use	Handling bodily fluids personal protective equipment use	Total personal protective equipment use score
**Cytotoxic**	Two pairs of gloves, gown, eye protection, N95 or P2 particulate filter mask					
**Median [IQR]**	10 [7, 16.5] *n* = 53	12 [6.75, 15] *n* = 66	13 [8.5, 15] *n* = 57		10 [7, 15] *n* = 72	
**Mean (SD)**						11.78 (4.83) *n* = 40
**Non-cytotoxic**	Single pair of gloves (chemotherapy or nitrile), gown, eye protection, N95 or P2 particulate filter mask					
**Median [IQR]**	11 [9, 16] *n* = 83	11 [9, 14] *n* = 95	10 [8, 13.5] *n* = 85		12 [10, 15] *n* = 116	
**Mean (SD)**						11.50 (3.95) *n* = 54

Table notes: Possible score range 0–20; score is the total of each of the four items of personal protective equipment for each handling activity stated in the table. The two missing responses were allocated the median response for that variable. The ‘total personal protective equipment use score’ is the mean of the medians for the four different handling activities for the participants who reported being involved in all four handling activities. IQR: interquartile range, *n*: frequency, SD: standard deviation.

### 3.7 Measures

The scale ‘knowledge about risk of exposure’ was found to have a low Cronbach alpha of 0.42 (best result of 0.44 when item five was removed, 0.39 for those who handled cytotoxic hazardous drugs), despite a Cronbach alpha of 0.64 with the nursing sample when the questionnaire was piloted (there were no maternity services at the pilot site). In this study, the median score was 10, with an interquartile range [9, 11] indicating low variability and a high total score in most of the participants’ responses. Therefore, the reliability of this scale with this sample was unacceptable (Boateng et al., 2018), and the results have not been used in any further analysis. The coefficient for each of the other scales used in this questionnaire was greater than 0.7, which means that the reliability of the other scales was acceptable (Pallant, 2020), as shown in the Supplementary Material.

#### 3.7.1 The theoretical predictor variables

Responses were analysed based on the number of complete responses to each question, and therefore each scale had a different sample size (*n*), as shown in Table 5.

**Table 5:** Descriptive statistics for each of the theoretical predictor variable scales.

Variable	*n*	Mean	Standard deviation	Number of items	Observed range	Possible range
Self-efficacy for using personal protective equipment	203	26.48	4.60	7	12–35	7–35
Barriers to using personal protective equipment	160	36.31	9.96	14	14–66	14–70
Perceived risk	158	21.02	3.28	7	13–35	7–35
Conflict of interest	156	16.33	5.09	6	6–30	6–30
Workplace safety climate	156	72.38	11.16	20	48–100	20–100

**Variable**	***n***	**Median**	**Interquartile range**	**Number of items**	**Observed range**	**Possible range**
Interpersonal modelling*	153	3.00	[1.67, 4.33]	3	0–5	0–5
Interpersonal norms*	156	1.37	[1.00, 2.00]	4	0–2	0–2

⁎ There was a ‘Does not apply’ option for this scale; therefore, the median instead of the total of the item scores was calculated. *n* = number of participants.

Each scale was plotted and evaluated for normality and outliers. The presence of two extreme outliers in the *perceived risk* scale was investigated and found to be probably correct for the participants. Participants reported high *self-efficacy for using personal protective equipment*. Most participants (181 [89.2 %]) agreed or strongly agreed that they were confident that they could use personal protective equipment correctly and were provided with the best available personal protective equipment (163 [80.3 %]). Moderate *barriers to using personal protective equipment* were reported. The most frequent overall responses agreed or strongly agreed with the statement “I am unsure which drugs are hazardous; therefore, I may unintentionally not use PPE” (119 [74.4 %]) and “PPE makes me feel too hot” (92 [57.5 %]).

Responses to items about ‘perceived risk’ had a mean of 3 (SD 0.47) on the 5-point Likert scale. In the *interpersonal modelling* items, the response to the third item to indicate that those who handled chemotherapy were observed to wear personal protective equipment when handling hazardous drugs "Always 100 %" of the time was high (74 [71.2 %]).

Participants reported a moderate *conflict of interest* between the well-being of the patient and the need to use safe handling and a high *workplace safety climate*.

#### 3.7.2 Test of the mediating effects of nurses and midwives, behaviour-specific cognitions, and the use of personal protective equipment

The predictor variables were normally distributed, except for the two interpersonal scales and the total ‘use of personal protective equipment’. To measure the strength of the correlation between the variables, Spearman's rho (ordinal), independent *t*-tests (binary), and the Mann-Whitney (binary and non-normal distribution) were used. The independent *t*-tests were conducted to compare the scale scores for nurses who did and did not work in cancer services.

There was a significant correlation between the score for *conflict of interest* and the number of years of work experience (*p*=.01). There was a statistically significant difference in scores for the *self-efficacy for using personal protective equipment* of nurses and midwives working in non-cancer services (mean 26.09, SD 4.47) compared to cancer services (mean=29.42, SD 4.54; *t* (203) = −3.38, *p*=.002, two-tailed); the magnitude of the differences in the means (mean difference = −3.33, 95 % confidence interval [−5.34, −1.31] was small (eta squared = 0.05). There was also a significant difference for ‘barriers to using personal protective equipment’ (Supplementary Material).

#### 3.7.3 Correlations among the theoretical predictor variables

To evaluate the relationship strength and direction between the theoretical predictor variables, bivariate correlations were calculated using Pearson's *r* correlation coefficients. There were significant negative relationships between *self-efficacy for using personal protective equipment* and *barriers to using personal protective equipment* (*r* = −0.363, *p* = <0.001), *workplace safety climate* and *barriers to using personal protective equipment, perceived risk* and *conflict of interest*, and between *self-efficacy for using personal protective equipment* and *conflict of interest* (*r* = −0.167, *p* = <0.05). There was a significant positive correlation between *self-efficacy for using personal protective equipment* and workplace safety climate (*r* = .576, *p* = <0.001), *barriers to using personal protective equipment* and both *perceived risk* and *conflict of interest*, and between *perceived risk* and *conflict of interest*, as shown in the Supplementary Material.

*Workplace safety climate* scores had a significant correlation with the *total use of personal protective equipment* for all cytotoxic hazardous drug handling activities. *Barriers to using personal protective equipment* negatively correlated significantly for all handling activities, except for handling bodily fluids (Supplementary Material).

In comparison, *self-efficacy for using personal protective equipment* correlated significantly with the *total use of personal protective equipment* for all non-cytotoxic hazardous drug handling activities except for handling bodily fluids, as shown in the Supplementary Material.

## 4 Discussion

In this study, we explored nurses’ and midwives’ knowledge and practices related to the safe handling of hazardous drugs. Participants correctly identified hazardous drugs approximately half of the time, and, although they reported being confident in using appropriate personal protective equipment, approximately 30 % reported never wearing it. Less experienced participants scored higher on the *conflict of interest* scale, and *workplace safety climate* was statistically significantly correlated to the *total use of personal protective equipment* when handling both cytotoxic and non-cytotoxic hazardous drugs.

### 4.1 Knowing that a hazard exists

The handling of hazardous drugs is widespread within healthcare services because these drugs are prescribed to treat a variety of medical conditions, such as epilepsy, hypertension, autoimmune diseases, and cancers (Victorian Therapeutics Advisory, 2021; van Huizen et al., 2024b). Consequently, there is a potential for occupational exposure wherever these drugs are handled, and this widespread risk can be unknown or underestimated by nurses and midwives (van Huizen et al., 2024b).

Participants scored an average of 54.1 % on the *‘*hazardous drug identification’ question. This result also aligns with the response that only 35.9 % of participants had received training about handling hazardous drugs. Consequently, because hazardous drugs are administered in almost all healthcare areas, in the future, it may be necessary for nurses and midwives who are at risk of occupational exposure to know that these drugs pose a risk and to be well-informed about what they could do to mitigate it. An example of the implementation of guidelines for safe handling, which includes all categories of hazardous drugs, has been reported in a multidisciplinary study by Vuelta-Arce et al. (2020). In the analytical phase, the authors identified the hazardous drugs in their hospital using the Spanish guidelines by the National Institute for Safety and Health at Work (Vuelta-Arce et al., 2020; Delgado Sánchez et al., 2016). They also investigated safe handling practices to mitigate the potential for exposure to people involved in handling hazardous drugs. In the development phase, the hazard was communicated, standard operating procedures were reviewed and updated, and the pharmacy service adopted measures that reduced the need for nurses to manipulate hazardous drugs (Vuelta-Arce et al., 2020). Vuelta-Arce et al. (2020) illustrated how the communication of the hazard and education of nurses and midwives needs to include all hazardous drugs and all the people who handle them. However, the literature is currently monopolised by antineoplastic drugs, which are a subset of hazardous drugs, and their handling by oncology nurses (van Huizen et al., 2024a).

The above Spanish and other similar guidelines refer to the universally -cited National Institute for Safety and Health *NIOSH list of hazardous drugs in healthcare settings*. In the most recent update of this list, the term ‘antineoplastic’ has been left out of both the document title and the Table 1 descriptor, because not all of the carcinogenic or probably carcinogenic drugs are antineoplastic (National Institute for Occupational Safety and Health, 2024). Additionally, an important recommendation is that risk management for drugs that are reproductive or developmental hazards has been suggested for all staff members, both female and male, and not just those who are pregnant or breastfeeding (National Institute for Occupational Safety and Health, 2023).

### 4.2 Knowledge

The results of the ‘knowledge about risk of exposure’ questions are comparable to other studies that used the same scale and reported data with populations of oncology nurses (Callahan et al., 2016; Crickman and Finnell, 2017; Polovich and Clark, 2012; Srisintorn et al., 2021). The high scores (means 10.16, 10.5, 10.9, median 10, respectively, out of a possible 12) indicate that participants generally knew about how exposure can occur. However, the results were significantly different from one study conducted in Jordan (Abu Sharour et al., 2021). In that study, Abu Sharour et al. (2021) reported that the results were unsatisfactory (mean 3.55), which was concerning, since safe handling precautions were positively correlated with greater knowledge. The authors discussed a lack of both undergraduate and workplace education and training and the absence of standards to evaluate nurses’ practice in cancer treatment centres in Jordan (Abu Sharour et al., 2021).

### 4.3 What nurses and midwives do to protect themselves

When handling hazardous drugs, gloves are an important item of personal protective equipment for protection against dermal absorption (Koller et al., 2018). In the current study, when handling hazardous drugs, approximately one third of participants reported that they never wore personal protective equipment when preparing, administering, or disposing of injectable and oral formulations. This is despite local guidelines recommending wearing two pairs (double) of disposable nitrile gloves that are long enough to cover wrist cuffs of a gown for handling cytotoxic drugs or contaminated bodily fluids and for handling all spills (Supplementary Material). A single pair of gloves is recommended to be worn for all other hazardous drugs (Victorian Therapeutics Advisory Group, 2021). In this study, less than a quarter of participants reported always wearing double gloves when administering cytotoxic hazardous drugs, and just over 60 % reported always wearing chemotherapy or standard nitrile gloves when administering non-cytotoxic hazardous drugs. In the literature, double gloves were reported to be worn by 18 % to 54 % of oncology nurses administering chemotherapy (Polovich and Martin, 2011; Silver et al., 2016; DeJoy et al., 2017; Menonna-Quinn et al., 2019; Graeve et al., 2017; Orujlu et al., 2016), and, in a Canadian study, one third of centres reported that nurses removed the outer pair of gloves before handling infusion pumps (Pinet et al., 2023). Together, these results indicate that best practices for wearing gloves, including double gloving for handling cytotoxic hazardous drugs, are not universally practiced. Double gloving has been recognised as a difficult practice to implement. It has a negative impact on tactile sensation, and double gloves are perceived to be uncomfortable or difficult to wear (Hennessy, 2016; Fazel et al., 2022).

A protective gown, which is long-sleeved, cuffed, fluid impervious, and fastens at the back, should be worn when manipulating all oral hazardous drugs; for handling parenteral, inhaled, or nebulised formulations; and when handling spills and bodily fluids (Victorian Therapeutics Advisory Group, 2021). Bodily fluids can contain a percentage of unchanged drug and active metabolites from prodrugs that are activated within the body (Cass et al., 2017; Szetela and Gibson, 2007). In some cases, these metabolites are more hazardous than the original drug (Szetela and Gibson, 2007). In this study, an impermeable gown was always worn when handling cytotoxic contaminated bodily fluids such as urine, faeces, vomit, and sweat by almost a third of participants. This was more than the inpatient and outpatient oncology nurses reported by Graeve et al. (2017) and Menonna-Quinn et al. (2019). In all of these studies, more than half of the respondents were not wearing a protective gown when handling bodily fluids, putting them at risk of exposure to hazardous drugs or their metabolites.

Safety glasses or a full-face shield and a P2 or N95 particulate filter fit tested mask, not a surgical mask, should be worn if there is a risk of aersolisation or inhalation of hazardous drugs and for handling all cytotoxic parental formulations (Victorian Therapeutics Advisory Group, 2021). In the current study, 22.7 % of nurses and midwives who administered cytotoxic hazardous drugs reported never wearing eye protection, and 12.1 % never wore a P2 or N95 particulate filter mask. The relatively low numbers who reported not wearing these items of personal protective equipment is likely related to the timing of the study in early 2023. At this time, in all clinical areas within the healthcare service, healthcare professionals were required to wear a mask due to the COVID-19 pandemic. Participants (99.5 %) reported they were expected to wear a P2 or N95 mask, and 95.0 % reported that they were available to use. A potential reason why some people reported not wearing a P2 or N95 mask could be because they were wearing a surgical mask. Surgical masks are loose-fitting and may be more comfortable to wear; however, they do not provide adequate protection against hazardous drug exposure (American Society of Health-System Pharmacists, 2018; Victorian Therapeutics Advisory Group, 2021; National Institute for Occupational Safety and Health, 2023). In contrast to this, in other published studies, eye protection and masks were reported as being the least used items when administering chemotherapy. In one study, when administrating hazardous drugs 76.6 % of inpatient and outpatient oncology nurses reported never wearing eye protection, and 44.7 % never wore a mask (Menonna-Quinn et al., 2019).

In the peer-reviewed literature about what nurses know and do about their occupational exposure to hazardous drugs, it is important to note that at the time of writing this article there were no studies found to compare the use of personal protective equipment by nurses or midwives when handling non-cytotoxic hazardous drugs.

### 4.4 Personal and workplace factors associated with risk reduction

The scales that were used to assess how peers can promote or discourage a plan of action through social pressure, encouragement, expectations and modelling behaviour (interpersonal norms and modelling) were reported to have a moderate effect. The responses for the first two of three modelling items were normally distributed. However, for the third item of the modelling scale, how often "nurses who handle chemotherapy" were observed to use personal protective equipment, the data were skewed to the right, with 71.2 % of participants reporting "100 % of the time". This observed practice, that nurses who handle chemotherapy more often wear personal protective equipment, was supported by the reported data for the theoretical predictive variables. There were significant differences between *self-efficacy for using personal protective equipment* (higher) and *barriers to using personal protective equipment* (lower) reported by nurses who worked in cancer services compared to those who did not (*p*<.05). Similarly, in a pilot study by Mitchell et al. (2021), oncology nurses were expected to know more about protecting themselves relative to other healthcare workers, according to interviewees. Unlike nurses working in cancer services, nurses and midwives in non-oncologic settings may not know that there is a potential for occupational exposure and what they should do to mitigate this risk (Eisenberg, 2018).

The number of years working as a nurse or midwife was negatively correlated with ‘conflict of interest’ (*p*<.001). This means that the less time a person had been working as a nurse or midwife, the higher the conflict of interest between the need to protect themselves by wearing personal protective equipment and caring for patients. Similarly, Lee et al. (2021) reported that higher clinical experience was associated with higher compliance with safe handling, and time pressure was associated with lower compliance. Novice healthcare professionals can find it difficult to cope with competing demands, and both time management and putting their own needs first can be difficult.

Similar to the study by Polovich and Clark (2012), there were significant relationships found among the theoretical predictor variables, except, in the current study, the relationship between perceived risk and the other theoretical predictor variables was in the opposite direction. The lower moderate perceived risk could be due to the population including nurses and midwives from all healthcare areas, as compared to only oncology nurses in the study by Polovich and Clark (2012).

*Workplace safety climate* was significantly correlated with wearing personal protective equipment for all cytotoxic hazardous drug handling activities and for non-cytotoxic hazardous drug handling activities, except for bodily fluids. When handling non-cytotoxic bodily fluids compared to cytotoxic ones, the number of participants who reported wearing a gown was low. The difference for this item of personal protective equipment may have been because of not identifying non-cytotoxic hazardous drugs as a hazard, despite a reported positive *workplace safety climate*, whereas the high level of reported glove wearing for handling bodily fluids aligns with expected standard precautions for infection control (National Health and Medical Research Council, 2019). A more positive *workplace safety climate* was a predictor of hazardous drug precaution use in other studies with oncology nurses (Srisintorn et al., 2021; Abu Sharour et al., 2021; Callahan et al., 2016) and, likewise, in a study from Japan, adherence to personal protective use for infection prevention was strongly influenced by organisational factors (Morioka et al., 2020).

### 4.5 Improving practice

To be successful, interventions to improve nurses’ and midwives’ knowledge and practice related to safely handling hazardous drugs require a multi-faceted, multidisciplinary approach (Vuelta-Arce et al., 2020). Interventions that have demonstrated improved knowledge and practice for nurses working in cancer services include situational influences, such as periodic audits, making personal protective equipment available where it is used, and professional development through video, a mobile phone application, online modules, workshops, and lectures (Crickman and Finnell, 2017; Hennessy and Dynan, 2014; Friese et al., 2019; Hojati et al., 2023; Jun and Kang, 2023; Keat et al., 2013; Nouri et al., 2021). Also, considering the widespread use of hazardous drugs throughout healthcare services, safety messages within electronic medical records and physically labelling these drugs as hazardous would improve communication of the hazard (Abu-Alhaija et al., 2023; Crickman and Finnell, 2017). Labelling could be either regulatory, so that it is mandated in law, or part of practice at an institutional level when receiving, unpacking, and storing hazardous drugs (National Institute for Occupational Safety and Health, 2023). Another way to do this, although non-cytotoxic hazardous drugs may be stored with non-hazardous drugs (American Society of Health-System Pharmacists, 2018), would be to store all hazardous drugs separately on easily identifiable shelving. Storing hazardous drugs separately would mean that nurses and midwives could easily identify which drugs are hazardous without spending time accessing information, and this environmental prompt could ‘nudge’ them to implement safe handling practices (Sant'Anna et al., 2021). Another possible intervention could be to use interpersonal influences. Signage could be used to encourage patients to speak up when personal protective equipment should be used and is not (Hennessy and Dynan, 2014).

### 4.6 Strengths and limitations

The strength of this study was the inclusion of nurses and midwives who handled hazardous drugs both inside and outside of cancer services. Although the diversity of hazardous drugs is mentioned in the literature, past research has been focused on the handling of antineoplastic drugs in cancer services.

The most important potential limitation is that the survey was based on the non-probability convenience sampling approach, and results were self-reported. Nurses and midwives who participated may be those who felt concerned about their safety or had a clinical nurse educator speak to them individually about the questionnaire. Unfortunately, this potential bias suggests that the low level of hazardous drug identification, the scores reported for the theoretical predictor variables, and the safe handling practices may be an under- or overestimation of these variables. Also, the data were collected from only one public healthcare service in Victoria, Australia, which limits the generalisability of the results. This was a cross-sectional study that identifies associations but not cause and effect.

One of the scale variables, ‘knowledge about risk of exposure’, did not achieve an acceptable reliability level (α=0.42) with this sample of nurses and midwives. Further revision of the questions and testing with this population is needed. Also, questions asked about crushing drugs and counting restricted drugs were not specific to hazardous drugs. From the results, it is unknown how frequently these practices involved hazardous drugs and, therefore, if these practices were definitively a potential source of occupational exposure.

## 5 Conclusion

We have added to the body of literature by including both nurses and midwives who work throughout the healthcare system and who may handle both cytotoxic and non-cytotoxic hazardous drugs.

Participants did not always recognise drugs as hazardous, and, although they were confident in using appropriate personal protective equipment, some reported never wearing it. Further, the recommended personal protective equipment was not always worn for all cytotoxic and non-cytotoxic hazardous drug formulations and drug handling activities. Multi-faceted, multidisciplinary interventions to improve both knowledge and practice for handling both cytotoxic and non-cytotoxic drugs by nurses and midwives may be needed. It is important that these interventions have a positive effect on workplace safety climate, which is correlated with the use of personal protective equipment.

## Funding

This research was supported by an Australian Government Research Training Program (RTP) Scholarship.

## CRediT authorship contribution statement

**Pheona van Huizen:** Writing – review & editing, Writing – original draft, Validation, Methodology, Investigation, Formal analysis, Data curation, Conceptualization. **Paul Wembridge:** Writing – review & editing, Validation, Methodology, Investigation, Conceptualization. **Philip L. Russo:** Writing – review & editing, Supervision. **Elizabeth Manias:** Writing – review & editing, Supervision. **Clifford J. Connell:** Writing – review & editing, Supervision.

## Declaration of competing interest

The authors declare that they have no known competing financial interests or personal relationships that could have appeared to influence the work reported in this paper.

## Appendix Supplementary materials

Image, application 1

Image, application 2

Image, application 3

Image, application 4

Image, application 5

Image, application 6

Image, application 7

## References

[bib0001] Abu Sharour L., Subih M., Bani Salameh A., Malak M. (2021). Predictors of chemotherapy safe-handling precautions and knowledge among a sample of Jordanian oncology nurses: a model-building approach. Workplace Health Saf..

[bib0002] Abu-Alhaija D., Bakas T., Shaughnessy E., Miller E. (2023). The factors that influence chemotherapy exposure among nurses: an integrative review. Workplace Health Saf..

[bib0003] American Society of Health-System Pharmacists (2018). ASHP guidelines on handling hazardous drugs. Am. J. Health-Syst. Pharm..

[bib54] American Society of Hospital Pharmacists [ASHP] (1990). ASHP technical assistance bulletin on handling cytotoxic and hazardous drugs. Amercian Journal of Hospital Pharmacy.

[bib0004] Asefa S., Aga F., Dinegde N., Demie T. (2021). Knowledge and practices on the safe handling of cytotoxic drugs among oncology nurses working at tertiary teaching hospitals in Addis Ababa, Ethiopia. Drug. Heal. Patient. Saf..

[bib0005] Boateng G., Neilands T., Frongillo E., Melgar-Quinonez H., Young S. (2018). Best practices for developing and validating scales for health, social, and behavioral research: a primer. Front Public Health..

[bib0006] Callahan A., Ames N., Manning M., Touchton-Leonard K., Yang L., Wallen G. (2016). Factors influencing nurses’ use of hazardous drug safe-handling precautions. Oncol Nurs Forum..

[bib0007] Cass Y., Connor T.H., Tabachnik A. (2017). Safe handling of oral antineoplastic medications: focus on targeted therapeutics in the home setting. J. Oncol. Pharm. Pract..

[bib0008] Crickman R., Finnell D. (2017). Chemotherapy safe handling. Limiting nursing exposure with a hazardous drug control program. Clin. J. Oncol. Nurs..

[bib55] Cronbach L. (1946). Response sets and test validity. Educat. Psychol. Manage..

[bib0009] Dejoy D., Smith T., Woldu H., Dyal M., Steege A., Boiano J. (2017). Effects of organizational safety practices and perceived safety climate on PPE usage, engineering controls, and adverse events involving liquid antineoplastic drugs among nurses. J. Occup. Env. Hyg..

[bib0010] Delafoy C., Roussy C., Hudon A., Cirtiu C., Caron N., Bussières J., Tanguay C. (2023). Canadian monitoring program of the surface contamination with 11 antineoplastic drugs in 122 centers. J. Oncol. Pharm. Pract..

[bib0011] Delgado Sánchez O., Guardino Solá X., Moreno Centeno E., Cercós Lleti A., Alonso Herreros J., Gaspar Carreño M., González-Haba Peña E. (2016).

[bib0012] Eisenberg S. (2018). Applying hazardous drug standards to antineoplastics used for ophthalmology surgery. AORN. J..

[bib0013] Faul F., Erdfelder E., Buchner A., Lang A. (2009). Statistical power analyses using G*Power 3.1: tests for correlation and regression analyses. Behav. Res. Method..

[bib0014] Fazel S.S., Keefe A., Shareef A., Palmer A.L., Brenner D.R., Nakashima L., Koehoorn M.W., Mcleod C.B., Hall A.L., Peters C.E. (2022). Barriers and facilitators for the safe handling of antineoplastic drugs. J. Oncol. Pharm. Pract..

[bib0015] Friese C., Yang J., Mendelsohn-Victor K., Mccullagh M. (2019). Randomized controlled trial of an intervention to improve nurses' hazardous drug handling. Oncol. Nurs. Forum..

[bib0016] Graeve C., Mcgovern P., Alexander B., Church T., Ryan A., Polovich M. (2017). Occupational exposure to antineoplastic agents. Workplace Health Saf..

[bib0017] Gruenewald B., Gilkey D. (2021). Hazardous drug exposure in healthcare. World Saf. J..

[bib0019] Hennessy K. (2016). The evolution of the safe handling of hazardous chemotherapy drugs. Oncol. Nurse-APN/PA..

[bib0020] Hennessy K., Dynan J. (2014). Improving compliance with personal protective equipment use through the model for improvement and staff champions. Clin J Oncol Nurs..

[bib0021] Hojati Z., Goudarzi F., Hasanvand S., Galehdar N., Birjandi M. (2023). The impact of training chemotherapy safety standards with a smartphone application on the knowledge, attitude, and performance of nurses. BMC Nurs..

[bib56] International Society of Oncology Pharmacy Practitioners Standards Committee [ISOPP] (2022). ISOPP standards for the safe handling of cytotoxics. Journal of Oncology Pharmacy Practice.

[bib0022] Jun E., Kang S. (2023). Effects of safe handling education on cognition, compliance and stress handling of antineoplastic drugs in clinical nurses. Nurs. Open..

[bib0023] Keat C., Sooaid N., Yun C., Sriraman M. (2013). Improving safety-related knowledge, attitude and practices of nurses handling cytotoxic anticancer drug: pharmacists' experience in a general hospital, Malaysia. Asian Pac. J. Cancer Prev..

[bib0024] Kennedy K., Vu K., Coakley N., Daley-Morris J., Forbes L., Hartzell R., Lessels D. (2023). Safe handling of hazardous drugs. J. Oncol. Pharm. Pract..

[bib0025] Koller M., Böhlandt A., Haberl C., Nowak D., Schierl R. (2018). Environmental and biological monitoring on an oncology ward during a complete working week. Toxicol. Let..

[bib0026] Lee H., Song J., Ahn J., Boo S. (2021). Factors influencing compliance with safe handling of antineoplastic agents among clinical nurses. Asian Oncol. Nurs..

[bib0027] Leilani A.U. (2023). MIMS Online.

[bib0028] Lusk S., Ronis D., Hogan M. (1997). Test of the health promotion model as a causal model of construction workers' use of hearing protection. Res. Nurs Health..

[bib0029] Menonna-Quinn D., Polovich M., Marshall B. (2019). Personal protective equipment: evaluating usage among inpatient and outpatient oncology nurses. Clin. J. Oncol. Nurs..

[bib0030] Mitchell P., Mcgovern P.M., Kirkhorn S. (2021). Improving questionnaires for medical surveillance of hazardous drug exposure: results of a pilot study. Workplace. Health. Saf..

[bib0031] Morioka S., Tajima T., Sugiki Y., Hayakawa K., Ohmagari N. (2020). Adherence to personal protective equipment use among nurses in Japanese tertiary care hospitals: what determines variability?. J. Hosp. Infect..

[bib0032] National Health and Medical Research Council (2019). *Australian Guidelines for the Prevention and Control of Infection in*.

[bib0033] National Institute for Occupational Safety and Health (2023). Managing hazardous drug exposures: information for healthcare settings.

[bib0034] National Institute for Occupational Safety and Health (2024). NIOSH list of hazardous drugs in healthcare settings, 2024.

[bib0035] Nouri A., Seyed Javadi M., Iranijam E., Aghamohammadi M. (2021). Improving nurses’ performance in the safe handling of antineoplastic agents: a quasi-experimental study. BMC Nurs.

[bib0036] Orujlu S., Habibzadeh H., Sakhvidi M., Hajaghazadeh M. (2016). Knowledge, attitude, and performance of oncology nurses handling antineoplastic drugs in hospitals of Urmia University, Iran. Int. J. Occup. Hyg..

[bib0037] Pallant J. (2020).

[bib0038] Pender N., Murdaugh C., Parsons M. (2015).

[bib0039] Pinet E., Langlais A., Chouinard A., Bussières J.-F., Tanguay C. (2023). National survey of safe handling of hazardous drugs in hospital settings: use of an innovative approach. J. Oncol. Pharm. Pract..

[bib0040] Pirot C., Benoist H., Saint-Lorant G. (2023). Impact of lack of knowledge on risk perception and protective practices of home nurses handling antineoplastic drugs. J. Oncol. Pharm. Pract..

[bib0041] Polovich M., Clark P. (2012). Factors influencing oncology nurses’ use of hazardous drug safe-handling precautions. Oncol Nurs Forum..

[bib0042] Polovich M., Martin S. (2011). Nurses’ use of hazardous drug-handling precautions and awareness of national safety guidelines. Oncol Nurs Forum..

[bib57] Qualtrics (2022). *Qualtrics*. Provo. https://www.qualtrics.com.

[bib0043] Raymond D., Hong O., Lusk S., Ronis D. (2006). Predictors of hearing protection use for Hispanic and non-Hispanic white factory workers. Res. Theory Nurs..

[bib0044] Sant'Anna A., Vilhelmsson A., Wolf A. (2021). Nudging healthcare professionals in clinical settings: a scoping review of the literature. BMC. Health. v. Res..

[bib0045] Sharma A., Minh Duc N., Luu Lam Thang T., Nam N., Ng S., Abbas K., Huy N., Marušić A., Paul C., Kwok J., Karbwang J., De Waure C., Drummond F., Kizawa Y., Taal E., Vermeulen J., Lee G., Gyedu A., To K., Verra M., Jacqz-Aigrain É., Leclercq W., Salminen S., Sherbourne C., Mintzes B., Lozano S., Tran U., Matsui M., Karamouzian M. (2021). A consensus-based checklist for reporting of survey studies (CROSS). J. Gen Intern Med..

[bib0046] Silver S., Steege A., Boiano J. (2016). Predictors of adherence to safe handling practices for antineoplastic drugs: a survey of hospital nurses. J. Occup Env. Hyg..

[bib0047] Srisintorn W., Geater A., Polovich M., Thongsuksai P. (2021). Factors influencing precautions against antineoplastic drug exposure among nurses and nurse assistants in Thailand. Int. Arch. Occup. Environ. Health..

[bib0048] Symons K., Ermer J. (2021). Australian Don't Rush to Crush. Therapeutic options For People Unable to Swallow Solid Oral Medicines.

[bib0049] Szetela A., Gibson D. (2007). How the new oral antineoplastics affect nursing practice: capecitabine serves to illustrate. AJN..

[bib0050] Van Huizen P., Russo P., Manias E., Kuhn L., Connell C. (2024). Knowledge and safe handling practices affecting the occupational exposure of nurses and midwives to hazardous drugs: a mixed methods systematic review. Int. J. Nurs. Stud..

[bib0051] Van Huizen P., Wembridge P., Russo P., Manias E., Connell C. (2024). The handling of hazardous medications by nurses and midwives: a retrospective cohort study. Int. J. Nurs. Stud..

[bib0053] Vuelta-Arce M., Chiapella-Micó C., Mestre-Prad M., Teixidó-Huerta X., Del Estal-Jiménez J., Rodríguez-Gías E., Guinovart-Alemany M. (2020). Comprehensive tackling to the safe handling of hazardous drugs: a multidisciplinary approach to clinical practice. Int. J. Occup Med Env. Health..

[bib0052] Victorian Therapeutics Advisory Group. 2021. *Victorian framework: handling of hazardous medicines* [Online]. VicTAG. Available: https://www.victag.org.au/VicTAG_Handling_of_Hazardous_Medicine_Framework_Nov_2021_Final.pdf [Accessed 25th May 2024].

