# Supplementary material for: Nurses’ and midwives’ knowledge and safe-handling practices related to hazardous drugs: A cross-sectional study
Source: Int J Nurs Stud Adv. 2025 Apr 14;8:100331. doi: 10.1016/j.ijnsa.2025.100331 (PMC12059394; doi:10.1016/j.ijnsa.2025.100331)
Supplement: Supplementary file 1 [file mmc1.docx]

**Variables influencing the safe handling practices of hazardous drugs by nurses and midwives**

**as they relate to the Health Promotion Model (revised)**

Indirectly

Behavioural outcome

Prior related behaviour; use of personal protective equipment, safe handling practices

Personal factors; gender, age, years of experience, knowledge, qualifications

Immediate competing demands (low control); patient needs, work and time pressures, staffing, forgetting and preferences (high control); comfort, sensation through gloves

Health-promoting behaviour;

safe hazardous drug handling practices, wearing appropriate personal protective equipment

Individual characteristics and experiences

Commitment to a plan of action

Perceived benefits of action

Perceived barriers to action

Perceived self-efficacy

Activity-related affect

Interpersonal influences; nurse unit manager, other nurses and midwives, patients

Situational influence; availability of personal protective and other equipment, training, workplace culture, policies, specialty healthcare area

Behaviour-specific cognitions and affect

Can be modified through interventions

**Figure 1** Adapted from Pender et al. (2015) with permission. The individual characteristics and experiences, behaviour-specific cognitions and affect, and the competing demands and preferences (in blue) were the data variables collected through the questionnaire.

Hazardous

non-cytotoxic

n=106

Cytotoxic

n=17

**Figure 2** Types of hazardous drugs handled by nurses and midwives (N=217, 1 missing response): 17 (7.8%) reported they handled cytotoxic drugs only, 93 (42.9%) reported handling hazardous non-cytotoxic drugs only and 106 (48.8%) nurses and midwives reported they handled both cytotoxic and non-cytotoxic hazardous drugs.
