# Supplementary material for: Nurses’ and midwives’ knowledge and safe-handling practices related to hazardous drugs: A cross-sectional study
Source: Int J Nurs Stud Adv. 2025 Apr 14;8:100331. doi: 10.1016/j.ijnsa.2025.100331 (PMC12059394; doi:10.1016/j.ijnsa.2025.100331)
Supplement: Supplementary file 2 [file mmc2.docx]

Questionnaire responses (n=310)

Blank (n=5)

Demographics (n=305)

Questions 1-7

Hazardous drugs identification (n=252)

Question 8

Cytotoxic (n=123)

Hazardous non-cytotoxic (n=199)

Both (n=106)

Demographics only (n=54)

No (n=32). Missing (n=3)

Yes (n=217). Demographics these participants

Missing (n=1)

Handle hazardous drugs?

Question 9

Type of hazardous drugs handled. Question 10

End questionnaire (n=89)

Question 12 (n=217)

Knowledge about risk of exposure

(missing=7)

Questions 13-16 (n=203)

Counting hazardous drugs

(missing n=1)

Self-efficacy for using PPE

Written policies

Availability PPE and COVID PPE

(missing 5)

Administer hazardous drugs? (n=177)

No (n=44)

Yes (n=152) Question 19-20

End questionnaire (n=1)

Prepare hazardous drugs? (n=196)

Questions 21-24 (n=178)

Preparation location (missing=20)

Remove packaging oral drugs

(missing=1)

Crushing device use

(missing=22)

Source information about crushing (missing=12)

Question 11 (n=217)

Education and training (missing=1)

Continue (n=26)

End questionnaire (n=18)

No (n=16)

Yes (n=161) Questions 26-28

Continue (n=15)

End questionnaire (n=1)

No (n=28)

Yes (n=136) Question 30-32

Continue (n=28)

End questionnaire (n=14)

No (n=20)

Continue (n=20)

End questionnaire (n=3)

Dispose of hazardous drugs? (n=164)

Handle bodily fluids? (n=163)

End questionnaire (n=12)

End questionnaire (n=1)

Questions 37-38 (n=163)

Spill kit availability and recent spill

Continue (n=160)

End questionnaire (n=14)

End questionnaire (n=7)

Yes (n=143) Question 34-36

Question 40 (n=159)

Perceived risk

Questions 41-44 (n=156)

Interpersonal modelling

Interpersonal norms

Conflict of interest

Workplace safety climate

Question 39 (n=160)

Barriers to using PPE

End questionnaire (n=3)

End questionnaire (n=1)

End questionnaire
