# Supplementary material for: Nurses’ and midwives’ knowledge and safe-handling practices related to hazardous drugs: A cross-sectional study
Source: Int J Nurs Stud Adv. 2025 Apr 14;8:100331. doi: 10.1016/j.ijnsa.2025.100331 (PMC12059394; doi:10.1016/j.ijnsa.2025.100331)
Supplement: Supplementary file 3 [file mmc3.pdf]

Recommendations for handling **cytotoxic** medicines						
**Route**	**Formulation**	**Disposable nitrile gloves; long enough to cover wrist cuffs of gown**	**Protective gown; long-sleeved, cuffed, fluid impervious, fastens at the back**	**Eye protection; safety glasses (over spectacles if wearing) or full face if increased risk of splash**	**Respiratory protection; fit tested mask with a P2 or N95 particulate filter, NOT a surgical mask**	**Spill kit**
Oral	Intact coated tablet/capsule	✓ two pairs				
	Uncoated tablet/capsule	✓ two pairs		✓ If risk of aersolisation or inhalation	✓ If risk of aersolisation or inhalation	
	Oral liquid	✓ two pairs	✓	✓ If risk of aersolisation or inhalation	✓ If risk of aersolisation or inhalation	
	Manipulated tablet/capsule (crushed or halved)	✓ two pairs	✓	✓ If risk of aersolisation or inhalation	✓ If risk of aersolisation or inhalation	
Parenteral	All parenteral formulations	✓ two pairs	✓	✓	✓	
Topical		✓ two pairs		✓ If risk of aersolisation or inhalation	✓ If risk of aersolisation or inhalation	
Recommendations for handling of hazardous **non-cytotoxic** and **reproductive hazardous** medicines; for reproductive hazardous medicines, personal protective equipment recommendations apply to staff who are trying to conceive, pregnant or breastfeeding						
Oral	Intact coated and uncoated tablet/capsule	✓ single pair				
	Oral liquid	✓ single pair	✓	✓ If risk of aersolisation or inhalation	✓ If risk of aersolisation or inhalation	
	Manipulated tablet/capsule (crushed or halved)	✓ single pair	✓	✓ If risk of aersolisation or inhalation	✓ If risk of aersolisation or inhalation	
Parenteral	All parenteral formulations	✓ single pair	✓	✓ If risk of aersolisation or inhalation	✓ If risk of aersolisation or inhalation	
Topical		✓ single pair		✓ If risk of aersolisation or inhalation	✓ If risk of aersolisation or inhalation	
Inhalation or nebulisation		✓ single pair	✓	✓	✓	
Recommendations for medicine **spills** and **bodily fluids**						
Medicine spill	All cytotoxic	✓ two pairs	✓	✓	✓	✓
	All other hazardous medicines	✓ two pairs	✓	✓	✓	✓
Bodily fluids (including spills and soiled linen)	Cytotoxic	✓ two pairs	✓	✓	✓	
	All other hazardous medicines	Standard infection control precautions as per organisational policies and procedures				

Victorian Therapeutics Advisory Group, 2021, addenda 21st September 2022 double gloves for the safe handling of cytotoxic and hazardous medicine spills and bodily fluids
