# Supplementary material for: Nurses’ and midwives’ knowledge and safe-handling practices related to hazardous drugs: A cross-sectional study
Source: Int J Nurs Stud Adv. 2025 Apr 14;8:100331. doi: 10.1016/j.ijnsa.2025.100331 (PMC12059394; doi:10.1016/j.ijnsa.2025.100331)
Supplement: Supplementary file 4 [file mmc4.docx]

**What nurses know about and do when handling hazardous drugs**

1. What is your gender?

- Woman (1)
- Man (2)
- Non-binary / gender diverse (3)
- My gender identity isn't listed. I identify as: (4) __________________________________________________
- Prefer not to say (5)

2. How old are you?

- 20-24 (1)
- 25-29 (2)
- 30-34 (3)
- 35-39 (4)
- 40-44 (5)
- 45-49 (6)
- 50-54 (7)
- 55-59 (8)
- 60 or older (9)

3. What qualifications have you completed? Select all that apply

- Diploma of Nursing (1)
- Bachelor of Nursing (2)
- Bachelor of Nursing (honours) (3)
- Bachelor of Midwifery (4)
- Graduate certificate of Nursing (5)
- Graduate diploma of Nursing (6)
- Master of Nursing (7)
- PhD (8)
- Other (9) __________________________________________________

4. How often do you work?

- Full-time (1)
- Part-time (2)
- Bank/casual (3)

5. Where do you work most often?

- Inpatient acute ward (1)
- Inpatient sub-acute ward (2)
- Day haematology or oncology (3)
- Outpatients and other same day settings (4)
- Residential care (5)
- Other (6) __________________________________________________

6 a. Which area/s of healthcare did you work in the last 7 days (last week)? Select all that apply.

- Breast and endocrine (1)
- Cancer services (2)
- Cardiology (3)
- Colorectal (4)
- Dermatology (5)
- Ear, nose and throat (6)
- Emergency (7)
- Endocrinology (8)
- Gastroenterology (9)
- General medicine (10)
- Geriatric medicine (11)
- Haematology (12)
- General/paediatric surgery (13)
- Infectious diseases (14)
- Intensive care services (15)
- Maternity services (16)
- Mental health services (17)
- Neurosciences (18)
- Orthopaedic (19)
- Paediatric (20)
- Palliative care (21)
- Plastic surgery (22)
- Rehabilitation (23)
- Renal (24)
- Residential aged care (25)
- Rheumatology (26)
- Respiratory (27)
- Special care nursery (28)
- Transition care (29)
- Upper gastrointestinal/bariatric/thoracic (30)
- Urology (31)
- Vascular (32)
- Other (33) __________________________________________________

6 b. Which location/s did you work in the last 7 days (last week)? Select all that apply

- Angliss (1)
- Blackburn (2)
- Box Hill (3)
- Healesville (4)
- Maroondah (5)
- Peter James (6)
- Wantirna (7)
- Other (8) __________________________________________________

6 c. What ward or healthcare area/s did you work at the most last week or for the week when you last worked?

________________________________________________________________

7. Years of nursing experience

- <1 year (1)
- 1-5 (2)
- 6-10 (3)
- 11-15 (4)
- 16-20 (5)
- 21-25 (6)
- 26 or more (7)

**A hazardous drug presents a danger to healthcare workers due to its inherent toxicity. Hazardous drugs are defined as having one or more of the following characteristics: they are carcinogenic, teratogenic or developmentally toxic, reproductively toxic, toxic to organs at low doses, or genotoxic (NIOSH definition).**

8. Which of these medications are classified as hazardous drugs? You will be shown the correct answers when you click on the bottom right arrow to continue.

|  | Yes (1) | No (0) | Don't know (3) |
| --- | --- | --- | --- |
| Acetylcysteine (Quiz_1) |  |  |  |
| Chloramphenicol (chlorsig) (Quiz_2) |  |  |  |
| Colchicine (Quiz_3) |  |  |  |
| Dutasteride (Quiz_4) |  |  |  |
| Fluconazole (Quiz_5) |  |  |  |
| Ibuprofen (Quiz_6) |  |  |  |
| Loperamide (Quiz_7) |  |  |  |
| Loratadine (Quiz_8) |  |  |  |
| Methotrexate (Quiz_9) |  |  |  |
| Oxytocin (Quiz_10) |  |  |  |
| Paracetamol (Quiz_11) |  |  |  |
| Paroxetine (Quiz_12) |  |  |  |
| Valproate (Quiz_13) |  |  |  |
| Warfarin (Quiz_14) |  |  |  |

**Handling hazardous drugs includes their preparation, administration, disposal, and cleaning up spills, as well as handling the bodily fluids (excreta) of patients that are taking these medications.**

9. Do you personally handle hazardous drugs at work?

- Yes (1)
- No (0)

*Skip To: End of Block If 9. Do you personally handle hazardous drugs at work? = No*

10. Which type of hazardous drugs to you handle? Select all that apply

- Cytotoxic (1)
- Hazardous non-cytotoxic (2)

**End of Block: Section 1B**

**Start of Block: Section 13**

Would you like the chance to win one of ten electronic $50 gift cards?

If you choose 'Yes', a separate page will open up for you to enter your email address. Your responses to the questionnaire will be stored separately from your email address so that your answers remain anonymous.

- Yes (4)
- No (5)

**End of Block: Section 13**

**Start of Block: Section 2 of 10**

11. Have you ever had any **training** about how to handle hazardous drugs?

- Yes (1)
- No (2)
- Unsure (3)

*Display This Question:*

*If 11. Have you ever had any training about how to handle hazardous drugs? = Yes*

11 b. When was the last time you completed training related to handling hazardous drugs?

12. Select one answer to each of the following elements about hazardous drug exposure for clinicians.

|  | True (1) | False (0) | Don't know (3) |
| --- | --- | --- | --- |
| Hazardous drugs can enter the body through inhalation (breathing it in) (Knowledge_1) |  |  |  |
| Hazardous drugs can enter the body through ingestion (from contamination on your hands) (Knowledge_2) |  |  |  |
| Hazardous drugs cannot enter the body through contact with contaminated surfaces (Knowledge_3) |  |  |  |
| Hazardous drugs can enter the body through contact with spills and splashes (Knowledge_4) |  |  |  |
| Hazardous drug gas and vapour in the air can enter the body through skin and mucous membranes (Knowledge_5) |  |  |  |
| Tablet forms of hazardous drugs do not have the potential to be absorbed though the skin (Knowledge_6) |  |  |  |
| Hazardous drugs in liquid form can be absorbed through the skin (Knowledge_7) |  |  |  |
| A surgical mask provides adequate protection from hazardous drug aerosols (Knowledge_8) |  |  |  |
| All types of gloves provide the same level of protection against hazardous drugs (Knowledge_9) |  |  |  |
| Hazardous drugs can more easily enter the body through damaged skin (Knowledge_10) |  |  |  |
| Alcohol hand rub (sanitiser) is as effective as soap and water in removing hazardous drug residue (Knowledge_11) |  |  |  |
| Hazardous drugs can be ingested (swallowed) through contaminated foods, beverages, or cosmetics (Knowledge_12) |  |  |  |

13. Removing oral medications from their bottles to count them, can produce residue and contaminate the work area. Before, during or at the end of your nursing shifts, do you ever

need to count medications by using a pill counting tray? For example, to count schedule 8 or schedule 11 drugs

- Yes (1)
- No (2)
- Other counting method used (4) __________________________________________________

**Self-efficacy**

14. Indicate your agreement with each of these statements about using personal protective equipment (PPE) when handling hazardous drugs

|  | Strongly agree (5) | Agree (4) | Neither agree nor disagree (3) | Disagree (2) | Strongly disagree (1) |
| --- | --- | --- | --- | --- | --- |
| I am confident that I can use PPE properly (Self-efficacy_1) |  |  |  |  |  |
| I am confident that I can protect myself from hazardous drug exposure (Self-efficacy_2) |  |  |  |  |  |
| I am given enough information on how to protect myself from hazardous drug exposure (Self-efficacy_3) |  |  |  |  |  |
| My manager takes the time and makes an effort to ensure I am protected (Self-efficacy_4) |  |  |  |  |  |
| Reusing PPE would make me feel less protected (Self-efficacy_5) |  |  |  |  |  |
| I am provided with the best available PPE (Self-efficacy_6) |  |  |  |  |  |
| My manager takes the time and makes an effort to ensure I am provided with proper fitting PPE (Self-efficacy_7) |  |  |  |  |  |

15. Does your workplace have written policies and guidelines for handling hazardous drugs?

- Yes (1)
- No (0)
- Don't know (3)

16. a. In your work area what personal protective equipment (PPE) is **AVAILABLE** for performing hazardous drug handling activities? Select all that apply

|  | Gloves (1) | Gowns/aprons (2) | Eye protection (3) | N95 or P2 mask (4) |
| --- | --- | --- | --- | --- |
| Available (1) |  |  |  |  |

16. b. Is there any PPE that you are currently required to wear in the clinical area because of the COVID-19 pandemic? If yes, please briefly list

________________________________________________________________

**Preparation includes reconstituting drugs, preparing intravenous (IV) infusions, measuring liquid medications, splitting and crushing tablets.**

17. Do you **PREPARE** medications that are classified as hazardous drugs?

- Yes (1)
- No (0)

*Display This Question:*

*If 17. Do you PREPARE medications that are classified as hazardous drugs? = Yes*

18. Do you wear PPE when **PREPARING** hazardous drugs? Select all that apply

- Yes, when preparing injectables (1)
- Yes, when preparing oral formulations (2)
- No, never (3)

*Display This Question:*

*If 18. Do you wear PPE when PREPARING hazardous drugs? Select all that apply = Yes, when preparing injectables*

*And 10. Which type of hazardous drugs to you handle? Select all that apply = Cytotoxic*

*Or 18. Do you wear PPE when PREPARING hazardous drugs? Select all that apply = Yes, when preparing oral formulations*

*And 10. Which type of hazardous drugs to you handle? Select all that apply = Cytotoxic*

19. Please indicate how often you use the following while **PREPARING** CYTOTOXIC hazardous dugs

|  | Always 100% (5) | 76-99% (4) | 51-75% (3) | 26-50% (2) | 1-25% (1) | Never 0% (0) |
| --- | --- | --- | --- | --- | --- | --- |
| Biological Safety Cabinet (ventilated, enclosed workspace) (Prep_cyto_1) |  |  |  |  |  |  |
| Closed system drug-transfer device (mechanically prevents leaks) (Prep_cyto_2) |  |  |  |  |  |  |
| Gloves labeled for chemotherapy (Prep_cyto_3) |  |  |  |  |  |  |
| Standard examination gloves (Prep_cyto_4) |  |  |  |  |  |  |
| Double layer of gloves (Prep_cyto_5) |  |  |  |  |  |  |
| Impermeable gown with a closed front and long sleeves with cuffs (Prep_cyto_6) |  |  |  |  |  |  |
| Other gown (eg. cloth or plastic disposable apron) (Prep_cyto_7) |  |  |  |  |  |  |
| Re-use disposable gowns (Prep_cyto_8) |  |  |  |  |  |  |
| Eye protection (Prep_cyto_9) |  |  |  |  |  |  |
| N95 or P2 mask (Prep_cyto_10) |  |  |  |  |  |  |

*Display This Question:*

*If 18. Do you wear PPE when PREPARING hazardous drugs? Select all that apply = Yes, when preparing injectables*

*And 10. Which type of hazardous drugs to you handle? Select all that apply = Hazardous non-cytotoxic*

*Or 18. Do you wear PPE when PREPARING hazardous drugs? Select all that apply = Yes, when preparing oral formulations*

*And 10. Which type of hazardous drugs to you handle? Select all that apply = Hazardous non-cytotoxic*

20. Please indicate how often you use the following while **PREPARING** NON-CYTOTOXIC hazardous dugs

|  | Always 100% (5) | 76-99% (4) | 51-75% (3) | 26-50% (2) | 1-25% (1) | Never 0% (0) |
| --- | --- | --- | --- | --- | --- | --- |
| Biological Safety Cabinet (ventilated, enclosed workspace) (Prep_non-cyto_1) |  |  |  |  |  |  |
| Closed system transfer device (mechanically prevents leaks) (Prep_non-cyto_2) |  |  |  |  |  |  |
| Gloves labeled for chemotherapy (Prep_non-cyto_3) |  |  |  |  |  |  |
| Standard examination gloves (Prep_non-cyto_4) |  |  |  |  |  |  |
| Double layer of gloves (Prep_non-cyto_5) |  |  |  |  |  |  |
| Impermeable gown with a closed front and long sleeves with cuffs. (Prep_non-cyto_6) |  |  |  |  |  |  |
| Other gown (eg. cloth or plastic disposable apron) (Prep_non-cyto_7) |  |  |  |  |  |  |
| Re-use disposable gowns (Prep_non-cyto_8) |  |  |  |  |  |  |
| Eye protection (Prep_non-cyto_9) |  |  |  |  |  |  |
| N95 or P2 mask (Prep_non-cyto_10) |  |  |  |  |  |  |

21. Where are hazardous drugs **PREPARED** in your workplace? Select all that apply.

- Onsite pharmacy (1)
- Drugs are delivered to the infusion area (prepared in an off-site location) (2)
- Specially designated medication room separate from the patient care area (3)
- Area within the patient treatment area/room (4)
- Medication trolley in hallway (5)
- Other (6) __________________________________________________
- Don't know ()

22. When you remove tablets or capsules from packaging, to administer oral medications, which do you do?

- Use aseptic non-touch technique, with no gloves (1)
- Use aseptic non-touch technique, with gloves (2)
- Standard handling of tablets, with gloves (3)
- Standard handling of tablets, without gloves (4)
- Other (5) __________________________________________________

23. Patients with swallowing difficulties, a percutaneous endoscopic gastrostomy (PEG) tube (feeding) or a nasogastric tube (NGT) in situ may require you to **CRUSH** medications before administration. How do you crush these medications?

- Using a mortar and pestle (1)
- Using a pill crushing device (2)
- In a syringe (3)
- Disperse in water (4)
- Other (5) __________________________________________________
- Not applicable (0)

24. Sometimes medications should not be crushed or broken (cut or split) because they have an enteric coating or they could be an occupational exposure risk to you. How do you find out if a medication should be CRUSHED by you? Select all that apply

- Ask a pharmacist (1)
- 'Don't rush to crush' handbook (2)
- MIMS (3)
- Ask a more senior nurse (4)
- I don't routinely check if a medication should or should not be crushed (5)
- Other (6) __________________________________________________
- Not applicable (0)

**Administration includes connecting and starting IV lines, giving injections, giving tablets, applying creams or eye drops, and accessing percutaneous endoscopic gastrostomy (PEG) tubes (feeding tubes) or nasogastric tubes (NGTs).**

25. Do you **ADMINISTER** medications that are hazardous drugs?

- Yes (1)
- No (0)

*Display This Question:*

*If 25. Do you ADMINISTER medications that are hazardous drugs? = Yes*

26. Do you wear PPE when **ADMINISTERING** hazardous drugs?

- Yes, when administering injectables (1)
- Yes, when administering oral formulations (2)
- No, never (3)

*Display This Question:*

*If 26. Do you wear PPE when ADMINISTERING hazardous drugs? = Yes, when administering injectables*

*And 10. Which type of hazardous drugs to you handle? Select all that apply = Cytotoxic*

*Or 26. Do you wear PPE when ADMINISTERING hazardous drugs? = Yes, when administering oral formulations*

*And 10. Which type of hazardous drugs to you handle? Select all that apply = Cytotoxic*

27. Please indicate how often you use the following while **ADMINISTERING** CYTOTOXIC hazardous drugs

|  | Always 100% (5) | 76-99% (4) | 51-75% (3) | 26-50% (2) | 1-25% (1) | Never 0% (0) |
| --- | --- | --- | --- | --- | --- | --- |
| Closed system transfer device (mechanically prevents leaks) (Admin_cyto_1) |  |  |  |  |  |  |
| Gloves labeled for use with chemotherapy (Admin_cyto_2) |  |  |  |  |  |  |
| Standard examination gloves (Admin_cyto_3) |  |  |  |  |  |  |
| Double layer of gloves (Admin_cyto_4) |  |  |  |  |  |  |
| Impermeable gowns with a closed front and long sleeves with cuffs. (Admin_cyto_5) |  |  |  |  |  |  |
| Other gown (eg. cloth or plastic disposable apron) (Admin_cyto_6) |  |  |  |  |  |  |
| Re-use disposable gowns (Admin_cyto_7) |  |  |  |  |  |  |
| Eye protection (Admin_cyto_8) |  |  |  |  |  |  |
| N95 or P2 mask (Admin_cyto_9) |  |  |  |  |  |  |

*Display This Question:*

*If 26. Do you wear PPE when ADMINISTERING hazardous drugs? = Yes, when administering injectables*

*And 10. Which type of hazardous drugs to you handle? Select all that apply = Hazardous non-cytotoxic*

*Or 26. Do you wear PPE when ADMINISTERING hazardous drugs? = Yes, when administering oral formulations*

*And 10. Which type of hazardous drugs to you handle? Select all that apply = Hazardous non-cytotoxic*

28. Please indicate how often you use the following while **ADMINISTERING** NON-CYTOTOXIC hazardous drugs

|  | Always 100% (5) | 76-99% (4) | 51-75% (3) | 26-50% (2) | 1-25% (1) | Never 0% (0) |
| --- | --- | --- | --- | --- | --- | --- |
| Closed system transfer device (mechanically prevents leaks) (Admin_non-cyto_1) |  |  |  |  |  |  |
| Gloves labeled for use with chemotherapy (Admin_non-cyto_2) |  |  |  |  |  |  |
| Standard examination gloves (Admin_non-cyto_3) |  |  |  |  |  |  |
| Double layer of gloves (Admin_non-cyto_4) |  |  |  |  |  |  |
| Impermeable gowns with a closed front and long sleeves with cuffs. (Admin_non-cyto_5) |  |  |  |  |  |  |
| Other gown (eg. cloth or plastic disposable apron) (Admin_non-cyto_6) |  |  |  |  |  |  |
| Re-use disposable gowns (Admin_non-cyto_7) |  |  |  |  |  |  |
| Eye protection (Admin_non-cyto_8) |  |  |  |  |  |  |
| N95 or P2 mask (Admin_non-cyto_9) |  |  |  |  |  |  |

**End of Block: Section 5**

**Start of Block: Section 6**

**Disposal includes discarding equipment, including finished IV infusions, and materials used when preparing or administering hazardous drugs.**

29. Do you **DISPOSE** of medications that are hazardous drugs and contaminated equipment (including finished intravenous infusions)?

- Yes (1)
- No (0)

*Display This Question:*

*If 29. Do you DISPOSE of medications that are hazardous drugs and contaminated equipment (including... = Yes*

30. Do you wear PPE when **DISPOSING** of hazardous drugs and contaminated equipment?

- Yes, when disposing of equipment and portions of unused drug used for injectables (1)
- Yes, when disposing of equipment and portions of unused drug used for oral formulations (2)
- No, never (3)

*Display This Question:*

*If 30. Do you wear PPE when DISPOSING of hazardous drugs and contaminated equipment? = Yes, when disposing of equipment and portions of unused drug used for injectables*

*And 10. Which type of hazardous drugs to you handle? Select all that apply = Cytotoxic*

*Or 30. Do you wear PPE when DISPOSING of hazardous drugs and contaminated equipment? = Yes, when disposing of equipment and portions of unused drug used for oral formulations*

*And 10. Which type of hazardous drugs to you handle? Select all that apply = Cytotoxic*

31. Please indicate how often you use the following when **DISPOSING** of CYTOTOXIC hazardous drugs and equipment

|  | Always 100% (5) | 76-99% (4) | 51-75% (3) | 26-50% (2) | 1-25% (1) | Never 0% (0) |
| --- | --- | --- | --- | --- | --- | --- |
| Gloves labeled for use with chemotherapy (Disposal_cyto_1) |  |  |  |  |  |  |
| Standard examination gloves (Disposal_cyto_2) |  |  |  |  |  |  |
| Double layer of gloves (Disposal_cyto_3) |  |  |  |  |  |  |
| Impermeable gowns with a closed front and long sleeves with cuffs. (Disposal_cyto_4) |  |  |  |  |  |  |
| Other gown (eg. cloth or plastic disposable apron) (Disposal_cyto_5) |  |  |  |  |  |  |
| Re-use disposable gowns (Disposal_cyto_6) |  |  |  |  |  |  |
| Eye protection (Disposal_cyto_7) |  |  |  |  |  |  |
| N95 or P2 mask (Disposal_cyto_8) |  |  |  |  |  |  |

*Display This Question:*

*If 30. Do you wear PPE when DISPOSING of hazardous drugs and contaminated equipment? = Yes, when disposing of equipment and portions of unused drug used for injectables*

*And 10. Which type of hazardous drugs to you handle? Select all that apply = Hazardous non-cytotoxic*

*Or 30. Do you wear PPE when DISPOSING of hazardous drugs and contaminated equipment? = Yes, when disposing of equipment and portions of unused drug used for oral formulations*

*And 10. Which type of hazardous drugs to you handle? Select all that apply = Hazardous non-cytotoxic*

32. Please indicate how often you use the following when **DISPOSING** of NON-CYTOTOXIC hazardous drugs and equipment

|  | Always 100% (5) | 76-99% (4) | 51-75% (3) | 26-50% (2) | 1-25% (1) | Never 0% (0) |
| --- | --- | --- | --- | --- | --- | --- |
| Gloves labeled for use with chemotherapy (1) |  |  |  |  |  |  |
| Standard examination gloves (2) |  |  |  |  |  |  |
| Double layer of gloves (3) |  |  |  |  |  |  |
| Impermeable gowns with a closed front and long sleeves with cuffs. (4) |  |  |  |  |  |  |
| Other gown (eg. cloth or plastic disposable apron) (5) |  |  |  |  |  |  |
| Re-use disposable gowns (6) |  |  |  |  |  |  |
| Eye protection (7) |  |  |  |  |  |  |
| N95 or P2 mask (8) |  |  |  |  |  |  |

33. Do you handle hazardous drug contaminated **BODILY FLUIDS** (excreta), this may include emptying bedpans and urinals or disposing of emesis bags, of patients who have recently been treated with hazardous drugs?

- Yes (1)
- No (0)

*Display This Question:*

*If 33. Do you handle hazardous drug contaminated BODILY FLUIDS (excreta), this may include emptying... = Yes*

34. Do you wear PPE when handling hazardous drug contaminated **BODILY FLUIDS** (excreta)?

- Yes (1)
- No, never (3)

*Display This Question:*

*If 34. Do you wear PPE when handling hazardous drug contaminated BODILY FLUIDS (excreta)? = Yes*

*And 10. Which type of hazardous drugs to you handle? Select all that apply = Cytotoxic*

35. Please indicate how often you use the following when handling **BODILY FLUIDS** (excreta) contaminated by CYTOTOXIC hazardous drugs

|  | Always 100% (5) | 76-99% (4) | 51-75% (3) | 26-50% (2) | 1-25% (1) | Never 0% (0) |
| --- | --- | --- | --- | --- | --- | --- |
| Gloves labeled for use with chemotherapy (1) |  |  |  |  |  |  |
| Standard examination gloves (2) |  |  |  |  |  |  |
| Double layer of gloves (3) |  |  |  |  |  |  |
| Impermeable gowns with a closed front and long sleeves with cuffs. (4) |  |  |  |  |  |  |
| Other gown (eg. cloth or plastic disposable apron) (5) |  |  |  |  |  |  |
| Re-use disposable gowns (6) |  |  |  |  |  |  |
| Eye protection (7) |  |  |  |  |  |  |
| N95 or P2 mask (8) |  |  |  |  |  |  |

*Display This Question:*

*If 34. Do you wear PPE when handling hazardous drug contaminated BODILY FLUIDS (excreta)? = Yes*

*And 10. Which type of hazardous drugs to you handle? Select all that apply = Hazardous non-cytotoxic*

36. Please indicate how often you use the following when handling **BODILY FLUIDS** (excreta) contaminated by NON-CYTOTOXIC hazardous drugs

|  | Always 100% (5) | 76-99% (4) | 51-75% (3) | 26-50% (2) | 1-25% (1) | Never 0% (0) |
| --- | --- | --- | --- | --- | --- | --- |
| Gloves labeled for use with chemotherapy (1) |  |  |  |  |  |  |
| Standard examination gloves (2) |  |  |  |  |  |  |
| Double layer of gloves (3) |  |  |  |  |  |  |
| Impermeable gowns with a closed front and long sleeves with cuffs. (4) |  |  |  |  |  |  |
| Other gown (eg. cloth or plastic disposable apron) (5) |  |  |  |  |  |  |
| Re-use disposable gowns (6) |  |  |  |  |  |  |
| Eye protection (7) |  |  |  |  |  |  |
| N95 or P2 mask (8) |  |  |  |  |  |  |

**End of Block: Section 6**

**Start of Block: Section 7**

37. Are hazardous drug (cytotoxic) **SPILL KITS** available in your work area?

- Yes (1)
- No (0)
- Don't know (3)

38. During the most recent **hazardous drug spill** in your workplace, did you use a hazardous drug (cytotoxic) spill kit to manage the spill?

- Yes (1)
- No (2)
- There has not been a spill that I know about (3)
- Not available (4)

**End of Block: Section 7**

**Start of Block: Section 8**

**Perceived barriers**

39. Indicate your level of agreement with each of the following statements.
Some reasons that I may not wear PPE regularly when handling hazardous drugs are:

|  | Strongly agree (5) | Agree (4) | Neither agree nor disagree (3) | Disagree (2) | Strongly disagree (1) |
| --- | --- | --- | --- | --- | --- |
| I don't think PPE is necessary (1) |  |  |  |  |  |
| I don't think PPE works (2) |  |  |  |  |  |
| I don't have the time to use PPE (3) |  |  |  |  |  |
| I was not trained to use PPE (4) |  |  |  |  |  |
| PPE is uncomfortable to wear (5) |  |  |  |  |  |
| PPE makes it harder to get the job done (6) |  |  |  |  |  |
| PPE is not always available (7) |  |  |  |  |  |
| Others around me don't use PPE (8) |  |  |  |  |  |
| There is no policy requiring PPE (9) |  |  |  |  |  |
| People would think I am overly cautious (10) |  |  |  |  |  |
| It is hard to get hazardous drug or cytotoxic designated PPE (11) |  |  |  |  |  |
| PPE is too expensive to use it all the time (12) |  |  |  |  |  |
| PPE makes me feel too hot (13) |  |  |  |  |  |
| I am unsure which medications are hazardous therefore I may unintentionally not use PPE (14) |  |  |  |  |  |

**End of Block: Section 8**

**Start of Block: Section 9**

**Perceived Risk**

40. Indicate your level of agreement with each of the following statements about the risks of hazardous drug exposure

|  | Strongly agree (5) | Agree (4) | Neither agree nor disagree (3) | Disagree (2) | Strongly disagree (1) |
| --- | --- | --- | --- | --- | --- |
| Exposure to hazardous drugs is a serious problem at work (1) |  |  |  |  |  |
| I am concerned about hazardous drug exposure at work and how it might affect my health (2) |  |  |  |  |  |
| Compared to co-workers, my chances of harm from hazardous drug exposure is lower (3) |  |  |  |  |  |
| If exposed to hazardous drugs, there is a real chance that I might experience bad health effects (4) |  |  |  |  |  |
| Hazardous drug exposure is not as harmful as some people claim (5) |  |  |  |  |  |
| Compared to other work-related health risks, hazardous drug exposure is less serious (6) |  |  |  |  |  |
| I am not worried about future negative health effects from hazardous drug exposure (7) |  |  |  |  |  |

**End of Block: Section 9**

**Start of Block: Section 10**

Interpersonal A

41. How often do you observe the following people wearing personal protective equipment (PPE) when handling hazardous drugs?

|  | Always 100% (5) | 76-99% (4) | 51-75% (3) | 26-50% (2) | 1-25% (1) | Never 0% (0) | Does not apply (8) |
| --- | --- | --- | --- | --- | --- | --- | --- |
| Nurses in your area (1) |  |  |  |  |  |  |  |
| Other nurses you know (2) |  |  |  |  |  |  |  |
| Nurses who handle chemotherapy (3) |  |  |  |  |  |  |  |

Interpersonal B

42. What is your perception about how important the following people think it is to wear PPE when handling hazardous drugs?

|  | Very important (2) | Sort of important (1) | Not at all important (0) | Does not apply (8) |
| --- | --- | --- | --- | --- |
| Nurses in your area (1) |  |  |  |  |
| Other nurses you know (2) |  |  |  |  |
| Your manager (3) |  |  |  |  |
| Your employer (4) |  |  |  |  |

**End of Block: Section 10**

**Start of Block: Section 11**

Conflict of interest

43. Indicate your level of agreement with each of the following statements

|  | Strongly agree (5) | Agree (4) | Neither agree nor disagree (3) | Disagree (2) | Strongly disagree (1) |
| --- | --- | --- | --- | --- | --- |
| Personal protective equipment (PPE) keeps me from doing my job to the best of my abilities (1) |  |  |  |  |  |
| Wearing PPE makes my patients worry (2) |  |  |  |  |  |
| Patient care often interferes with my being able to comply with using precautions (3) |  |  |  |  |  |
| I cannot always use safe handling precautions because patients' needs come first (4) |  |  |  |  |  |
| Sometimes I have to choose between wearing PPE and the immediate care of my patients (5) |  |  |  |  |  |
| Wearing PPE makes my patients feel uncomfortable (6) |  |  |  |  |  |

**End of Block: Section 11**

**Start of Block: Section 12**

**This is the last question.**

Workplace safety culture

44. Indicate your level of agreement with these statements regarding safety in your work place

|  | Strongly agree (5) | Agree (4) | Neither agree nor disagree (3) | Disagree (2) | Strongly disagree (1) |
| --- | --- | --- | --- | --- | --- |
| Chemotherapy gloves are readily accessible in my work area (1) |  |  |  |  |  |
| Impermeable gowns with a closed front and long sleeves with cuffs are readily available in my area (2) |  |  |  |  |  |
| I perceive that the protection of workers from occupational exposure to hazardous drugs is a high priority with management where I work (3) |  |  |  |  |  |
| On my unit, all reasonable steps are taken to minimise hazardous job tasks (4) |  |  |  |  |  |
| Employees are encouraged to become involved in health and safety matters (5) |  |  |  |  |  |
| Managers of my unit do their part to ensure employees' protection from occupational exposure to hazardous drugs (6) |  |  |  |  |  |
| My job duties do not often interfere with me being able to follow hazardous drug safe handling precautions (7) |  |  |  |  |  |
| I always have enough time in my work day to follow hazardous drug safe handling precautions (8) |  |  |  |  |  |
| On my unit, unsafe work practices are corrected by my manager (10) |  |  |  |  |  |
| My manager talks to me about safe work practices (11) |  |  |  |  |  |
| I have had the opportunity to be properly trained to use PPE so that I can protect myself from hazardous drug exposures (12) |  |  |  |  |  |
| Employees are taught to be aware of and to recognise potential health hazards at work (13) |  |  |  |  |  |
| In my work area, I have access to policies and procedures regarding safety (14) |  |  |  |  |  |
| My work area is kept clean (15) |  |  |  |  |  |
| My work area is not cluttered (16) |  |  |  |  |  |
| My work area is not crowded (17) |  |  |  |  |  |
| There is minimal conflict between employees within my work area (18) |  |  |  |  |  |
| The members of my work area support one another (19) |  |  |  |  |  |
| In my work area, there is open communication between the manager and staff (20) |  |  |  |  |  |
| In my work area we are expected to comply with safe handling policies and procedures (21) |  |  |  |  |  |

Comments

Any comments that you would like to make about your responses

________________________________________________________________

**End of Block: Section 12**
