# Supplementary material for: Nurses’ and midwives’ knowledge and safe-handling practices related to hazardous drugs: A cross-sectional study
Source: Int J Nurs Stud Adv. 2025 Apr 14;8:100331. doi: 10.1016/j.ijnsa.2025.100331 (PMC12059394; doi:10.1016/j.ijnsa.2025.100331)
Supplement: Supplementary file 5 [file mmc5.docx]

**Supplementary material - results**

The use of engineering and personal protective equipment hazard controls by nurses and midwives handling cytotoxic and non-cytotoxic hazardous drugs, *n* (%)

|  | **How often** | **Preparation** | | **Administration** | | **Disposal** | | **Handle bodily fluids** | |
| --- | --- | --- | --- | --- | --- | --- | --- | --- | --- |
|  |  | **Cytotoxic**  ***N*=53**  ***n* (%)** | **Non-cytotoxic**  ***N*=83**  ***n* (%)** | **Cytotoxic**  ***N*=66**  ***n* (%)** | **Non-cytotoxic**  ***N*=95**  ***n* (%)** | **Cytotoxic**  ***N*=57**  ***n* (%)** | **Non-cytotoxic**  ***N*=85**  ***n* (%)** | **Cytotoxic**  ***N*=72**  ***n* (%)** | **Non-cytotoxic**  ***N*=116**  ***n* (%)** |
| **Biological safety cabinet** | Always 100% | 15 (28.3) | 15 (18.1) |  |  |  |  |  |  |
|  | 76-99% | 6 (11.3) | 6 (7.2) |  |  |  |  |  |  |
|  | 51-75% | 3 (5.7) | 4(4.8) |  |  |  |  |  |  |
|  | 26-50% | 5 (9.4) | 5 (6.0) |  |  |  |  |  |  |
|  | 1-25% | 1 (1.9) | 6(7.2) |  |  |  |  |  |  |
|  | Never 0% | 23 (43.4) | 47 (56.6) |  |  |  |  |  |  |
| **Closed system transfer device** | Always 100% | 12^*^ (23.1) | 11 (13.3) | 27 (40.9) | 22^*^ (23.4) |  |  |  |  |
|  | 76-99% | 5 (9.4) | 3 (3.6) | 6 (9.1) | 7 (7.4) |  |  |  |  |
|  | 51-75% | 2 (3.8) | 8 (9.6) | 1 (1.5) | 5 (5.3) |  |  |  |  |
|  | 26-50% | 6 (11.3) | 5 (6.0) | 4 (6.1) | 2 (2.1) |  |  |  |  |
|  | 1-25% | 0 (0.0) | 2 (2.4) | 3 (4.5) | 6 (6.3) |  |  |  |  |
|  | Never 0% | 27 (50.9) | 53 (63.9) | 25 (37.9) | 52 (54.7) |  |  |  |  |
| **Chemotherapy gloves** | Always 100% | 22 (41.5) | 19 (22.9) | 31 (47.0) | 19 (20.0) | 29 (50.9) | 10 (11.8) | 26 (36.1) | 21 (18.1) |
|  | 76-99% | 5 (9.4) | 1 (1.9) | 4 (6.1) | 3 (3.2) | 2 (3.5) | 1 (1.2) | 4 (5.6) | 0 (0.0) |
|  | 51-75% | 2 (3.8) | 6 (7.2) | 7 (10.6) | 2 (2.1) | 1 (1.8) | 3 (3.5) | 1 (1.4) | 5 (4.3) |
|  | 26-50% | 2 (3.8) | 4 (4.8) | 0 (0.0) | 1 (1.1) | 0 (0.0) | 4 (4.7) | 0 (0.0) | 1 (0.9) |
|  | 1-25% | 2 (3.8) | 0 (0.0) | 3 (4.5) | 3 (3.2) | 4 (7.0) | 5 (5.9) | 6 (8.3) | 4 (3.4) |
|  | Never 0% | 20 (37.7) | 53 (63.9) | 21 (31.8) | 67 (70.5) | 21 (36.8) | 62 (72.9) | 35 (48.6) | 85 (73.3) |
| **Standard gloves** | Always 100% | 30 (56.6) | 39 (47.0) | 33 (50.0) | 39 (41.1) | 27 (47.4) | 38 (44.7) | 52 (72.2) | 97^*^ (84.3) |
|  | 76-99% | 6 (11.3) | 14 (16.9) | 3 (4.5) | 15 (15.8) | 3 (5.3) | 13 (15.3) | 4 (5.6) | 8 (6.9) |
|  | 51-75% | 4 (7.5) | 15 (18.1) | 5 (7.6) | 9 (9.5) | 6 (10.5) | 7 (8.2) | 2 (2.8) | 2 (1.7) |
|  | 26-50% | 2 (3.8) | 5 (6.0) | 2 (3.0) | 8 (8.4) | 3 (5.3) | 10 (11.8) | 1 (1.4) | 2 (1.7) |
|  | 1-25% | 0 (0.0) | 6 (7.2) | 3 (4.5) | 9 (9.5) | 0 (0.0) | 5 (5.9) | 0 (0.0) | 0 (0.0) |
|  | Never 0% | 11 (20.8) | 4 (4.8) | 20 (30.3) | 15 (15.8) | 18 (31.6) | 12 (14.1) | 13 (18.1) | 6 (5.2) |
| **Double gloves** | Always 100% | 12 (22.6) | 9 (10.8) | 16 (24.2) | 8^*^ (8.5) | 12 (21.1) | 8 (9.4) | 20 (27.8) | 16^*^ (13.9) |
|  | 76-99% | 4 (7.5) | 4 (4.8) | 5 (7.6) | 4 (4.2) | 5 (8.8) | 4 (4.7) | 5 (6.9) | 6 (5.2) |
|  | 51-75% | 6 (11.3) | 6 (7.2) | 3 (4.5) | 4 (4.2) | 0 (0.0) | 4 (4.7) | 5 (6.9) | 5 (4.3) |
|  | 26-50% | 3 (5.7) | 0 (0.0) | 3 (4.5) | 3 (3.2) | 2 (3.5) | 3 (3.5) | 4 (5.6) | 4 (3.4) |
|  | 1-25% | 2 (3.6) | 9 (10.8) | 1 (1.5) | 7 (7.4) | 4 (7.0) | 1 (1.2) | 3 (4.2) | 9 (7.8) |
|  | Never 0% | 26 (49.1) | 55 (66.3) | 38 (57.6) | 68 (71.6) | 34 (59.6) | 65 (76.5) | 35 (48.6) | 75 (64.7) |
| **Impermeable gown** | Always 100% | 20 (37.7) | 15 (18.1) | 26 (39.4) | 9 (9.5) | 25 (43.9) | 5 (5.9) | 22 (30.6) | 10^*^ (8.7) |
|  | 76-99% | 2 (3.8) | 5 (6.0) | 5 (7.6) | 4 (4.2) | 3 (5.3) | 3 (3.5) | 6 (8.3) | 5 (4.3) |
|  | 51-75% | 5 (9.4) | 3 (3.6) | 5 (7.6) | 4 (4.2) | 5 (8.8) | 3 (3.5) | 2 (2.8) | 7 (6.0) |
|  | 26-50% | 3 (5.7) | 4 (4.8) | 2 (3.0) | 3 (3.2) | 2 (3.5) | 5 (5.9) | 4 (5.6) | 10 (8.6) |
|  | 1-25% | 4 (7.5) | 5 (6.0) | 2 (3.0) | 11 (11.6) | 4 (7.0) | 9 (10.6) | 5 (6.9) | 15 (12.9) |
|  | Never 0% | 19 (35.8) | 51 (61.4) | 26 (39.4) | 64 (67.4) | 18 (31.6) | 60 (70.6) | 33 (45.8) | 68 (58.6) |
| **Other gown** | Always 100% | 8 (15.1) | 7 (8.4) | 11 (16.7) | 7 (7.4) | 8 (14.0) | 6 (7.1) | *12 (16.7) | 14^*^ (12.2) |
|  | 76-99% | 3 (5.7) | 5 (6.0) | 5 (7.6) | 7 (7.4) | 3 (5.3) | 4 (4.7) | 3 (4.2) | 5 (4.3) |
|  | 51-75% | 3 (5.7) | 7 (8.4) | 0 (0.0) | 4 (4.2) | 3 (5.3) | 5 (5.9) | 3 (4.2) | 8 (6.9) |
|  | 26-50% | 4 (7.5) | 2 (2.4) | 5 (7.6) | 4 (4.2) | 4 (7.0) | 3 (3.5) | 5 (6.9) | 16 (13.8) |
|  | 1-25% | 3 (5.7) | 8 (9.6) | 5 (7.6) | 7 (7.4) | 1 (1.8) | 4 (4.7) | 5 (6.9) | 10 (8.6) |
|  | Never 0% | 32 (60.4) | 54 (65.1) | 40 (60.6) | 66 (69.5) | 38 (66.7) | 63 (74.1) | 43 (59.7) | 62 (53.4) |
| **Re-use gown** | Always 100% | 3 (5.7) | 5 (6.0) | 5 (7.6) | 2 (2.1) | 5 (8.8) | 1 (1.2) | *4 (5.6) | 5^*^ (4.3) |
|  | 76-99% | 0 (0.0) | 1 (1.2) | 2 (3.0) | 1 (1.1) | 0 (0.0) | 2 (2.4) | 1 (1.4) | 0 (0.0) |
|  | 51-75% | 1 (1.9) | 1 (1.2) | 0 (0.0) | 0 (0.0) | 2 (3.5) | 1 (1.2) | 1 (1.4) | 1 (0.9) |
|  | 26-50% | 1 (1.9) | 0 (0.0) | 3 (4.5) | 1 (1.1) | 2 (3.5) | 3 (3.5) | 0 (0.0) | 2 (1.7) |
|  | 1-25% | 0 (0.0) | 3 (3.6) | 1 (1.5) | 2 (2.1) | 0 (0.0) | 2 (2.4) | 1 (1.4) | 4 (3.4) |
|  | Never 0% | 48 (90.6) | 73 (88.0) | 55 (83.3) | 89 (93.7) | 48 (84.2) | 76 (89.4) | 64 (88.9) | 103 (88.8) |
| **Eye protection** | Always 100% | 22 (41.5) | 25 (30.1) | 27 (40.9) | 20 (21.1) | 25 (43.9) | 14 (16.5) | *21 (29.2) | 28 (24.1) |
|  | 76-99% | 3 (5.7) | 4 (4.8) | 3 (4.5) | 9 (9.5) | 6 (10.5) | 8 (9.4) | 5 (6.9) | 6 (5.2) |
|  | 51-75% | 4 (7.5) | 11 (13.3) | 11 (16.7) | 11 (11.6) | 7 (12.3) | 6 (7.1) | 10 (13.9) | 12 (10.3) |
|  | 26-50% | 4 (7.5) | 9 (10.8) | 4 (6.1) | 10 (10.5) | 4 (7.0) | 9 (10.6) | 7 (9.7) | 10 (8.6) |
|  | 1-25% | 8 (15.1) | 8 (9.6) | 6 (9.1) | 16 (16.8) | 5 (8.8) | 12 (14.1) | 9 (12.5) | 18 (15.5) |
|  | Never 0% | 12 (22.6) | 26 (31.3) | 15 (22.7) | 29 (30.5) | 10 (17.5) | 36 (42.4) | 19 (26.4) | 41 (35.3) |
| **N95 or P2 mask** | Always 100% | 43 (81.1) | 70 (84.3) | 52^*^ (80.0) | 78 (82.1) | 49 (86.0) | 68 (80.0) | 61 (84.7) | 98 (84.5) |
|  | 76-99% | 3 (5.7) | 2 (2.4) | 2 (3.0) | 4 (4.2) | 2 (3.5) | 5 (5.9) | 2 (2.8) | 6 (5.2) |
|  | 51-75% | 1 (1.9) | 4 (4.8) | 1 (1.5) | 2 (2.1) | 1 (1.8) | 1 (1.2) | 3 (4.2) | 0 (0.0) |
|  | 26-50% | 2 (3.8) | 0 (0.0) | 1 (1.5) | 3 (3.2) | 1 (1.8) | 2 (2.4) | 1 (1.4) | 2 (1.7) |
|  | 1-25% | 0 (0.0) | 1 (1.2) | 1 (1.5) | 0 (0.0) | 0 (0.0) | 0 (0.0) | 0 (0.0) | 1 (0.9) |
|  | Never 0% | 4 (7.5) | 6 (7.2) | 8 (12.1) | 8 (8.4) | 4 (7.0) | 9 (10.6) | 5 (6.9) | 8 (6.9) |

^*^1 missing response, N: sample size, n: frequency. The mean and standard deviations are calculated for the recommended individual item of personal protective equipment for each handling activity, chemotherapy specific or other nitrile gloves both provide protection (Dugheri et al., 2022).

Dugheri, S., Mucci, N., Arcangeli, G., Trevisani, L., Squillaci, D., Bucaletti, E., Cappelli, G., & Mini, E. (2022). An update on permeation of protective medical gloves by antineoplastic drugs. *Sigurnost*, *64*(4), 341-357. <https://doi.org/10.31306/s.64.4.2>
