# Supplementary material for: Nurses’ and midwives’ knowledge and safe-handling practices related to hazardous drugs: A cross-sectional study
Source: Int J Nurs Stud Adv. 2025 Apr 14;8:100331. doi: 10.1016/j.ijnsa.2025.100331 (PMC12059394; doi:10.1016/j.ijnsa.2025.100331)
Supplement: Supplementary file 6 [file mmc6.docx]

**Supplementary material – Statistical analysis**

**Table 1**

Correlations between the nurses’ and midwives’ characteristics, the theoretical predictor variables and the use of personal protective equipment

| **Correlations** | | | **Self-efficacy for using personal protective equipment** | **Barriers to using personal protective equipment** | **Perceived risk** | **Conflict of interest** | **Workplace safety climate** | **Use of personal protective equipment - cytotoxic** | **Use of personal protective equipment - non-cytotoxic** |
| --- | --- | --- | --- | --- | --- | --- | --- | --- | --- |
| **Spearman’s rho** | **Nursing/midwifery years of experience** | Correlation coefficient  Sig. (2-tailed)  *n* | .031  .663  203 | -.128  .108  160 | 033  .676  159 | -.215  .007**  156 | .065  .420  156 | -.004  .979  40 | -.007  .958  54 |
| **Spearman’s rho** | **Level of education** | Correlation coefficient  Sig. (2-tailed)  *n* | .019  .788  203 | -.029  .719  160 | -.005  0.946  159 | -.109  .175  156 | .055  .496  156 | .093  .566  40 | -.055  .692  54 |
| **Independent samples t-test** | **Work in cancer services** | No (*n*)  No Mean (SD)  Yes (*n*)  Yes Mean (SD)  Mean difference  Confidence interval  Sig (2-tailed)  Cohen’s d  Variance explained by location | 179  26.09 (4.47)  24  29.42 (4.54)  -3.33  [-5.34, -1.31]  .002*  -.742  5.4% | 141  37.20 (9.85)  19  29.74 (8.33)  1.15  [2.78, 12.14]  .002*  9.693  0.8% | 140  25.41 (4.17)  19  26.53 (3.49)  -1.11  [-3.09, 0.87]  .268  4.095  0.8% | 137  16.50 (5.10)  19  15.05 (4.93)  1.17  [-1.01, 3.91]  .245  5.083  0.9% | 137  71.24 (10.92)  19  80.58 (9.55)  -3.54  [-14.55, -4.13]  <.001  10.766  7.5% |  |  |
| **Mann Witney** | **Work in cancer services** | No (*n*)  No Median  Yes (*n*)  Yes Median  Sig  Mann-Witney U  Standardised test stat (z)  Effect size (r) |  |  |  |  |  | 31  11.00  9  14.50  .106  190.0  1.637  0.26 | 45  11.50  9  13.25  .137  266.5  1.487  0.20 |

*Correlation is significant at the 0.05 level (2-tailed), **Correlation is significant at the 0.01 level (2-tailed), *n*: sample size, SD: standard deviation, Sig: significant

**Table 2**

Correlations among the theoretical predictor variables

|  | **Pearson correlation *r*** | **Self-efficacy for using personal protective equipment** | **Barriers to using personal protective equipment** | **Perceived risk** | **Conflict of interest** |
| --- | --- | --- | --- | --- | --- |
| **Barriers to using personal protective equipment** | Correlation  Sig (2-tailed)  *n* | -.363**  <.001  160 |  |  |  |
| **Perceived risk** | Correlation  Sig (2-tailed)  *n* | -.069  .389  158 | .323**  <.001  158 |  |  |
| **Conflict of interest** | Correlation  Sig (2-tailed)  *n* | -.167*  .038  156 | .713**  <.001  156 | .266**  <.001  155 |  |
| **Workplace safety climate** | Correlation  Sig (2-tailed)  *n* | .576**  <.001  156 | -.429**  <.001  156 | -.160*  .047  155 | -.303**  <.001  156 |

*Correlation is significant at the 0.05 level (2-tailed), **Correlation is significant at the 0.01 level (2-tailed),

*n*: sample size, sig: significant

**Table 3**

Correlations between **cytotoxic** personal protective equipment use and the theoretical predictor variables

|  | **Spearman’s**  **Correlation *rho*** | **Preparation** | **Administration** | **Disposal** | **Handling bodily fluids** | **Total personal protective equipment use** |
| --- | --- | --- | --- | --- | --- | --- |
| **Self-efficacy for using personal protective equipment** | Correlation  Sig (2-tailed) | .321*  .043 | .299  .061 | .309  .052 | .203  .209 | .315*  .048 |
| **Barriers to using personal protective equipment** | Correlation  Sig (2-tailed) | -.510**  <.001 | -.520**  <.001 | -.566**  <.001 | -.263  .106 | -.546**  <.001 |
| **Perceived risk** | Correlation  Sig (2-tailed) | .322*  .046 | .278  .087 | .264  .104 | .113  .492 | .319*  .048 |
| **Conflict of interest** | Correlation  Sig (2-tailed) | -.188  .258 | -.246  .136 | -.245  .137 | -.114  .495 | -.258  .118 |
| **Workplace safety climate** | Correlation  Sig (2-tailed) | .431**  .007 | .471**  .003 | .516**  <.001 | .324*  .047 | .481**  .002 |

*Correlation is significant at the 0.05 level (2-tailed), **Correlation is significant at the 0.01 level (2-tailed), sig: significant

**Table 4**

Correlations between **non-Cytotoxic** personal protective equipment use and the theoretical predictor variables

|  | **Spearman’s**  **Correlation *rho*** | **Preparation** | **Administration** | **Disposal** | **Handling bodily fluids** | **Total personal protective equipment use** |
| --- | --- | --- | --- | --- | --- | --- |
| **Self-efficacy for using personal protective equipment** | Correlation  Sig (2-tailed) | .378**  .005 | .280*  .041 | .405**  .002 | .092  .508 | .366**  .007 |
| **Barriers to using personal protective equipment** | Correlation  Sig (2-tailed) | -.332*  .014 | -.240  .081 | -.218  .114 | .087  .531 | -.245  .074 |
| **Perceived risk** | Correlation  Sig (2-tailed) | .167  .228 | .067  .632 | .049  .723 | -.083  .552 | .097  .486 |
| **Conflict of interest** | Correlation  Sig (2-tailed) | -.139  .316 | -.150  .279 | -.140  .311 | .163  .240 | -.121  .383 |
| **Workplace safety climate** | Correlation  Sig (2-tailed) | .391**  .003 | .372**  .006 | .303*  .026 | .077  .579 | .375**  .005 |

*Correlation is significant at the 0.05 level (2-tailed), **Correlation is significant at the 0.01 level (2-tailed), sig: significant
