# Supplementary material for: Nurses’ and midwives’ knowledge and safe-handling practices related to hazardous drugs: A cross-sectional study
Source: Int J Nurs Stud Adv. 2025 Apr 14;8:100331. doi: 10.1016/j.ijnsa.2025.100331 (PMC12059394; doi:10.1016/j.ijnsa.2025.100331)
Supplement: Supplementary file 7 [file mmc7.docx]

**Validity**

To assess the content validity, individual items were reviewed for content, clarity and comprehensiveness. Six experts in hazardous drug handling, with clinical expertise in oncology, pharmacy, nursing, education, and questionnaire design, determined the extent to which the individual items represented the concepts of interest.

Communication with the experts occurred via email, which included a document explaining the research question, aims and conceptual definitions of each of the subscale concepts, as shown in Table 1. The ‘relevance’ and ‘clarity’ scales were added to each item of the ‘expert’s version’ of the Qualtrics questionnaire. The experts were encouraged to suggest revisions for an item if they thought that it was inconsistent with the conceptual definitions, inaccurately represented the content domain or required re-wording for clarity.

**Table 1** The research aims and conceptual definitions of each of the subscale concepts

| **Concept** | **Theoretical definition** | **Operational definition (Instrument items)** |
| --- | --- | --- |
| **Objective: To explore the knowledge of, and practices related to the safe handling of hazardous drugs among nurses working in oncology, as well as nurses and midwives working in non-oncological healthcare settings** | | |
| Identify hazardous drugs | Hazardous drugs are carcinogenic, teratogenic or have other developmental toxicity, reproductively toxic, toxic to organs at low doses, genotoxic or similar to a drug that has the above characteristics | Section 1 Q8: Yes/No/Don’t know  Correct answer = 1; range 0-14 |
| Knowledge about risk of exposure | Hazardous drug exposure is through direct contact or from surface and air contamination leading to dermal and mucosal absorption, inhalation or ingestion | Modified ‘Chemotherapy exposure knowledge’ scale.  Section 2 Q11: True/False/Don’t know; Correct answer = 1; range 0-12 |
| Preparation practice | Dispensing, crushing or breaking tablets or opening capsules. Practices when reconstituting drugs, preparing intravenous infusions, measuring liquid drugs or dispersing tablets | Practices with oral drugs Section 3 Q15-17  Use of personal protective and other equipment Section 4 Q20-23  Always - 5/76-99% - 4/51-75% - 3/26-50% - 2/1-25% - 1/Never - 0 |
| Administration practice | Connecting and starting intravenous lines, administering injections or oral medications, applying creams and eye drops, and accessing percutaneous endoscopic gastrostomy (PEG) tubes or nasogastric tubes (NGT) | Use of personal protective equipment Section 5 Q24-27  Always - 5/76-99% - 4/51-75% - 3/26-50% - 2/1-25% - 1/Never - 0 |
| Disposal practice | Discarding equipment, including finished IV infusions, and equipment or packaging used when preparing or administering hazardous drugs | Use of personal protective equipment Section 6 Q28-29  Always - 5/76-99% - 4/51-75% - 3/26-50% - 2/1-25% - 1/Never - 0 |
| Other practices:  Use of spill kit  Counting tablets  Handling excreta | Tasks that can put the nurse or midwife at an increased risk of exposure including cleaning spills, removing tablets from packaging for counting and handling hazardous drug contaminated bodily fluids (excreta) | Section 8 Q33: Yes/No/There has not been a spill that I know about/Not available  Section 3 Q18: Yes/No, Text entry  Section 7 Q30-31  Always - 5/76-99% - 4/51-75% - 3/26-50% - 2/1-25% - 1/Never - 0 |
| **Objective: To describe controls of risk that are available to support nurses and midwives to implement best practice when handling hazardous drugs in healthcare settings** | | |
| Training provided | Education prior to starting work in a setting, and regular training throughout employment | Section 2 Q10: Yes/No/Maybe |
| Policies and guidelines | The presence and availability of documents that outline best and/or expected practice | Section 3 Q13: Yes/No/Don’t Know |
| To minimise risk from hazardous drug preparation | Surface contamination is common when hazardous drugs are prepared; a central appropriately equipped location can reduce risk | Section 3 Q14: Locations where preparation occurs selected, can be more than one |
| Availability of personal protective equipment | Personal protective equipment needs to be available where it will be used | Section 3 Q19 Checklist |
| Availability of spill kit | Spill kits need to be available where there may be a spill | Section 8 Q32 Yes/No/Don’t know |
| **Objective: To explore any attitudes or factors that affect the use of controls of risk for nurses and midwives at both an organisational and personal level** | | |
| Perceived self-efficacy (for using personal protective equipment) | Judgement of personal capability to perform an action, including skills and confidence | ‘Self-efficacy scale’ section 3 Q12: Strongly agree - 5, Agree - 4, Neither agree – 3 or disagree - 2, Strongly disagree – 1 |
| Perceived barriers (for using personal protective equipment) | Perceptions about resistance to, or difficulties of undertaking a behaviour | ‘Barriers to using personal protective equipment’ scale Section 8 Q34: Strongly agree - 5, Agree - 4, Neither agree – 3 or disagree - 2, Strongly disagree – 1 |
| Perceived risk | Risk perception of the likelihood, susceptibility, and severity of an outcome | Section 9 Q35: Strongly agree - 5, Agree - 4, Neither agree – 3 or disagree - 2, Strongly disagree – 1 |
| Interpersonal influences | Effect of considerations about others’ behaviours, beliefs, or attitudes | ‘Interpersonal norms’ and ‘Interpersonal modelling’ Section 10 Q36: Never – 0, Sometimes – 1, About half the time – 2, Usually - 3, Does not apply - 0; Range 0-9. Q37: Not at all important - 0, Sort of important - 1, Very important – 2, Does not apply |
| Perceived conflict of interest | A conflict between the well-being of the patient and the need to use personal protective equipment and safe handling practices | ‘Conflict of interest scale’ Section 11 Q38: Strongly agree - 5, Agree - 4, Neither agree – 3 or disagree - 2, Strongly disagree – 1 |
| Workplace safety culture | Organisational dimensions including the availability of safety equipment, management support, absence of job hindrances, feedback and training, cleanliness and orderliness, minimal conflict and good communication | ‘The workplace safety climate’ Section 12 Q39: Strongly agree - 5, Agree - 4, Neither agree – 3 or disagree - 2, Strongly disagree – 1 |

The number of items in the instrument with an item-level content validity (I-CVI) score of at least 0.83 for relevance and clarity was 91 out of 105; 4 scored 0.67 or 0.5 for relevance, and 10 scored 0.67 or 0.5 for clarity.

The initial version of the questionnaire included items about wearing personal protective equipment when handling hazardous drugs, as a broad classification. However, expert feedback suggested that different personal protective equipment may be worn depending on whether a hazardous drug was classified as cytotoxic or non-cytotoxic. This led to the major revision of the questionnaire.

To address the feedback, a question was added at the beginning of the questionnaire to have participants specify their involvement in handling cytotoxic, non-cytotoxic or both types of hazardous drugs. A decision was made to not include reproductively hazardous drugs because it was unknown whether nurses or midwives would be able to identify these as a separate hazard category. The questionnaire was then further modified to present only the relevant questions based on each participant’s answer to this initial question. After implementing these major changes, the questionnaire items with a content validity index score for relevance or clarity of 0.8 or less also underwent review.

An extra item was added to the ‘perceived barriers’ scale to include the concept that nurses may not be aware that a drug is hazardous “I am unsure which drugs are hazardous therefore I may unintentionally not use PPE”. Also, the availability of personal protective equipment was simplified into one question, removing duplication. Likewise, if nurses responded that they wore personal protective equipment, the type of personal protective equipment and amount of time that it was worn were consolidated into the same question for preparation and administration items.

For the first interpersonal items the scale was changed to match the other scales for personal protective equipment use, to avoid confusion caused by having multiple different scales. The order for the second interpersonal scale items were reversed to match the other scales in terms of the most positive answer being first. An item was removed from the workplace safety climate scale “I usually do not have too much to do so that I can follow chemotherapy safe handling precautions” following feedback that it was too similar to another item.

After implementing the feedback by editing the text of some items, sometimes significantly modifying the content and wording, the two experts who initially provided the most ‘not clear’ (1) or ‘somewhat clear’ (2) scores were contacted again. The changes made were discussed and the modified questionnaire items reassessed for relevance and clarity.

**Reliability**

The internal consistency and test-retest reliability of the scales in the questionnaire were also evaluated (Pallant, 2020). Cronbach’s alpha coefficient was used (n=52), and a value above 0.7 was accepted to indicate homogeneity of the items (Pallant, 2020). Test-retest reliability evaluated the degree to which the instructions and items in the questionnaire elicited consistent interpretations and responses when administered across different time points under similar conditions (Norwood, 2010).

To assess the test-retest reliability, nurses (there were no midwives at the pilot site) were asked to complete the questionnaire twice, with the second administration occurring approximately two weeks after the first. The two-week interval was to strike a balance between mitigating potential memory or mood effects while accounting for broader changes over time due to confounding factors (Mc Gillicuddy et al., 2016). Intraclass correlation coefficients (ICCs) were calculated (n=10). ICCs closer to 1.0 indicate a more reliable scale, with a minimum ICC of 0.8 recommended as an acceptable threshold (Polit, 2014). Unfortunately, although there were 52 responses to the first completion of the pilot questionnaire, only ten people completed the questionnaire twice. The ICCs were calculated; however it is acknowledged that this is a small sample size.

The established internal consistency from the components of the questionnaire used in previous studies, alongside this study are summarized in Table 2. The Cronbach alpha for the ‘Chemotherapy exposure knowledge’ and ‘Perceived risk’ scales were both below 0.7. Despite this, it was decided to retain both of these items and potentially split the results into two groups: those working exclusively with cytotoxic hazardous drugs and those working with non-cytotoxic drugs. This approach, if it was necessary, would determine if the scales were only reliable for the population of nurses working in cancer services.

**Table 2** Reliability of instruments incorporated into the questionnaire

| **Scale** | **Internal consistency**  **(Original study)** | **Polovich & Clark, 2012** | **Pilot this study** |
| --- | --- | --- | --- |
| Chemotherapy exposure Knowledge | α=0.63  (Geer et al., 2006) | α=0.7  Test-retest reliability 0.35 | α=0.64  Test-retest reliability  0.62 |
| Self-efficacy for using personal protective equipment | α=0.88  (Geer et al., 2006) | α=0.79  Test-retest reliability 0.7 | α=0.82 (n=31)  Test-retest reliability  0.89 |
| Barriers to using personal protective equipment | α=0.87  (Geer et al., 2006) | α=0.88  Test-retest reliability 0.72 | α=0.88 (n=30)  Test-retest reliability  0.52 |
| Perceived risk | α=0.94  (Geer et al., 2007) | α=0.72  Test-retest reliability 0.78 | α=0.54  Test-retest reliability  0.60 |
| Interpersonal influence in the workplace | Theta=0.75 (norms); 0.68 (modelling); 0.73 (support)  (McCullagh et al., 2002) | α=0.8  Test-retest reliability 0.92 | α=0.83 modelling  α=0.85 norms  Test-retest reliability  0.71 modelling  0.60 norms |
| Conflict of interest (Healthcare worker questionnaire) | α=0.72  (Gershon et al., 1995) | α=0.89  Test-retest reliability 0.7 | α=0.87  Test-retest reliability  0.44 |
| Workplace safety climate | α=0.71-0.84 (6 dimensions) Factor analysis  (Gershon et al., 2000) | α=0.93 (21 items)  Test-retest reliability 0.86 | α=0.91 (n=29)  Test-retest reliability  0.42 |

α = Cronbach’s alpha

**Table 3** Reliability statistics of the different theoretical predictor variable scales from the questionnaire

| **Variable** | **Sample measurement item** | **Cronbach’s alpha** |
| --- | --- | --- |
| Knowledge about risk of exposure | Hazardous drugs can enter the body through contact with spills and splashes | α=0.42 |
| Self-efficacy for using personal protective equipment | I am confident that I can protect myself from hazardous drug exposure | α=0.79 |
| Barriers to using personal protective equipment | PPE is uncomfortable to wear | α=0.87 |
| Perceived risk | I am concerned about hazardous drug exposure at work and how it might affect my health | α=0.70 |
| Interpersonal modelling | How often do you observe the following people wearing personal protective equipment (PPE) when handling hazardous drugs? Nurses in your area | α=0.80 |
| Interpersonal norms | What is your perception about how important the following people think it is to wear PPE when handling hazardous drugs? Other nurses you know | α=0.89 |
| Conflict of interest | Sometimes I have to choose between wearing PPE and the immediate care of my patients | α=0.85 |
| Workplace safety climate | Impermeable gowns with a closed front and long sleeves with cuffs are readily available in my area | α=0.88 |

α: Cronbach’s alpha, PPE: Personal protective equipment.

**References**

Geer, L., Anna, D., Curbow, B., Diener-West, M., van Wendel de Joode, B., & Mitchell, C. (2007). Survey assessment of worker dermal exposure and underlying behavioral determinants. *Journal of Occupational and Environmental Hygiene*, *4*(11), 809-820. <https://doi.org/https://doi.org/10.1080/15459620701612722>

Geer, L., Curbow, B., Anna, D., Lees, P., & Buckley, T. (2006). Development of a questionnaire to assess worker knowledge, attitudes and perceptions underlying dermal exposure. *Scandinavian journal of work, environment & health*, *32*(3), 209-218. <https://doi.org/10.5271/sjweh.1001>

Gershon, R., Karkashian, C., Grosch, J., Murphy, L., Escamilla-Cejudo, A., Flanagan, P., Bernacki, E., Kasting, C., & Martin, L. (2000). Hospital safety climate and it’s relationship witth safe work practices and workplace exposure incidents. *American Journal of Infection Control*, *28*(3), 211-221. <https://doi.org/10.1067/mic.2000.105288>

Gershon, R., Vlahov, D., Felknor, S., Vesley, D., Johnson, P., Delclos, G., & Murphy, L. (1995). Compliance with universal precautions among health care workers at three regional hospitals *American Journal of Infection Control*, *23*(4), 225-236. <https://doi.org/10.1016/0196-6553(95)90067-5>

Mc Gillicuddy, A., Kellya, M., Sweeney, C., Carmichael, A., Crean, A., & Sahm, L. (2016). Modification of oral dosage forms for the older adult: An Irish prevalence study. *International Journal of Pharmaceutics*, *510*(1), 386-393. <https://doi.org/10.1016/j.ijpharm.2016.06.056>

McCullagh, M., Lusk, S., & Ronis, D. (2002). Factors influencing use of hearing protection among farmers. *Nursing Research*, *51*(1), 33-39. <https://doi.org/10.1097/00006199-200201000-00006>

Norwood, S. (2010). *Research essentials. Foundations for evidence-based practice*. Pearson.

Pallant, J. (2020). *SPSS survival manual: A step by step guide to data analysis using IBM SPSS* (7th ed.). Allen & Unwin.
